# Enriched Environment Ameliorates Cerebral Ischemia–Reperfusion Injury via Dopamine–H_2_S Axis-Mediated Dual Mitophagy Activation

**DOI:** 10.3390/antiox15010052

**Published:** 2025-12-30

**Authors:** Bao Zhou, Haocheng Qin, Pengkun Yang, Na Ren, Lu Sun, Zhengran Ding, Zhong He, Shuai Zhang, Zijian Hua, Ya Zheng, Ce Li, Shenyi Kuang, Yulian Zhu, Kewei Yu

**Affiliations:** 1 Department of Rehabilitation Medicine, Huashan Hospital, Fudan University, Shanghai 200040, China; 23211220070@m.fudan.edu.cn (B.Z.);; 2 National Center for Neurological Disorders, Shanghai 200040, China; 3 Department of Neurology, Huashan Hospital, Fudan University, Shanghai 200040, China

**Keywords:** cerebral ischemia/reperfusion injury, hydrogen sulfide, mitophagy, oxidative stress, apoptosis, enriched environment

## Abstract

Cerebral ischemia–reperfusion injury triggers mitochondrial dysfunction and oxidative stress, exacerbating neuronal apoptosis. Emerging evidence highlights hydrogen sulfide (H_2_S) as a gasotransmitter modulating redox balance, autophagy, and apoptosis. This study investigates the neuroprotective mechanisms of Enriched Environment (EE) against ischemic injury, focusing on mitochondrial dynamics and H_2_S-mediated pathways. Using MCAO mice and OGD/R-treated SH-SY5Y neurons, interventions targeting H_2_S synthesis, hypoxia-inducible factor 1-alpha (HIF-1α), and mitophagy were implemented. Behavioral, histological, and molecular analyses demonstrated EE significantly improved neurological outcomes, suppressed apoptosis, and attenuated oxidative damage (reduced MDA, elevated MnSOD/glutathione). Mechanistically, EE enhanced mitophagy via dual pathways: canonical PINK1/parkin-mediated mitochondrial clearance, corroborated by transmission electron microscope and LC3B/parkin colocalization, and non-canonical HIF-1α/BNIP3L axis activation. Transcriptomic and Co-immunoprecipitation (Co-IP) data revealed EE upregulated endogenous H_2_S biosynthesis post-injury by promoting dopamine-induced calcium influx, which activated calmodulin-dependent signaling to stimulate cystathionine β-synthase/γ-lyase expression. Pharmacological blockade of H_2_S synthesis or HIF-1α abolished mitochondrial protection, confirming H_2_S as a central mediator. Notably, H_2_S exerted antiapoptotic effects by restoring mitochondrial integrity through synergistic mitophagy activation and oxidative stress mitigation. These findings propose a novel neuroprotective cascade: EE-induced dopaminergic signaling potentiates H_2_S production, which coordinates PINK1/parkin and HIF-1α/BNIP3L pathways to eliminate dysfunctional mitochondria, thereby preserving neuronal homeostasis. This study elucidates therapeutic potential of EE via H_2_S-driven mitochondrial quality control, offering insights for ischemic brain injury intervention.

## 1. Introduction

Stroke is an acute cerebrovascular disorder characterized by neuronal injury and focal neurological deficits. According to the latest data from 2021, ischemic stroke accounts for approximately 65.3% (62.4–67.7) of all stroke cases worldwide [1]. Current clinical management primarily involves intravenous thrombolysis, mechanical thrombectomy, and antithrombotic therapy, aiming to promptly restore cerebral blood flow perfusion [2]. However, successful recanalization is often accompanied by cerebral ischemia–reperfusion injury (CIRI), in which oxidative stress serves as a pivotal pathological mechanism [3].

During ischemia, the deprivation of oxygen and glucose disrupts neuronal metabolism, resulting in excessive accumulation of reactive oxygen species (ROS). Upon reperfusion, the abrupt reintroduction of oxygen triggers a burst of free radical chain reactions, leading to a transient surge in ROS levels. This ROS overload induces a series of cytotoxic events, including lipid peroxidation, protein modification, and DNA damage, thereby further exacerbating oxidative stress [4]. Given its central role in CIRI pathogenesis, oxidative stress has become a major therapeutic target. Overcoming the limitations of existing treatments and developing novel strategies to alleviate CIRI remains of critical clinical and theoretical importance.

Mitochondria are central targets of oxidative stress. As the primary intracellular source of ROS, mitochondrial dysfunction exacerbates ROS overproduction under pathological conditions [5]. To maintain cellular homeostasis, mitophagy—a selective form of autophagy that eliminates damaged mitochondria—plays an essential neuroprotective role. However, excessive ROS can in turn impair autophagic processes, resulting in defective mitochondrial clearance. This forms a vicious cycle of ROS accumulation and mitophagy dysfunction, which further amplifies neuronal injury in CIRI [6]. Moreover, mitochondrial dynamics are significantly disrupted during CIRI, characterized by enhanced mitochondrial fission and impaired fusion. These alterations reduce the reserve of functional mitochondria and aggravate cellular energy failure [7]. Collectively, these findings highlight the potential of targeting mitochondrial homeostasis as a promising therapeutic avenue for mitigating CIRI-induced neuronal damage [8].

Our research group has long been devoted to investigating non-pharmacological interventions as promising strategies for promoting neurological functional recovery [9,10,11,12], with the aim of providing mechanistic insights and translational implications for post-stroke rehabilitation. The enriched environment (EE) is a non-pharmacological behavioral intervention that enhances neuroplasticity and cognitive function by providing multisensory stimulation, social interaction, and voluntary physical activity. This concept was first proposed by Donald Hebb [13]. To date, EE has demonstrated considerable therapeutic potential in the field of neurorehabilitation. Studies have shown that EE may exert its beneficial effects through the upregulation of neurotrophic factors such as brain-derived neurotrophic factor (BDNF), vascular endothelial growth factor (VEGF), and nerve growth factor (NGF), thereby promoting neurogenesis, angiogenesis, and synaptic plasticity [13,14,15]. In addition, accumulating evidence indicates that EE may confer neuroprotection by modulating cell death pathways—including apoptosis, pyroptosis, and ferroptosis—as well as by improving cerebral blood flow [16]. Our previous work demonstrated that EE significantly enhanced autophagic flux and mitophagy, contributing to improved functional recovery after cerebral ischemia in mice [9]. However, whether EE exerts its protective effects by modulating the oxidative stress–mitophagy axis remains unclear, and the potential regulatory role of EE in mitochondrial function via gasotransmitters has not been systematically investigated.

Hydrogen sulfide (H_2_S), recognized as the third endogenous gasotransmitter following nitric oxide (NO) and carbon monoxide (CO), has recently gained attention for its antiapoptotic and antioxidant roles in the cardiovascular and nervous systems [17,18]. H_2_S is capable of directly scavenging ROS and alleviating oxidative stress. Moreover, it has been shown to stabilize mitochondrial function by modulating mitochondrial membrane potential, influencing components of the electron transport chain, regulating energy metabolism-related enzymes, and promoting mitochondrial fusion [19]. However, the role of H_2_S in mitophagy regulation and the underlying molecular mechanisms remain incompletely understood [19,20,21], particularly in the context of neuronal function. Compared to traditional antioxidants, H_2_S reacts more rapidly with ROS, yet its clinical application is limited by a short biological half-life and a narrow therapeutic concentration window. Therefore, exploring feasible non-pharmacological strategies, such as EE, to enhance endogenous H_2_S production—and investigating its role as a regulatory hub linking oxidative stress to mitophagy—may offer novel insights for the treatment of cerebral ischemia and stroke recovery.

Notably, dopamine, a critical neurotransmitter and metabolic modulator in the brain, has emerged as a potential therapeutic target in the context of cerebral ischemic injury [22]. Previous studies have indicated that dopamine may indirectly regulate the expression of hydrogen sulfide (H_2_S)-producing enzymes, such as cystathionine β-synthase (CBS) and cystathionine γ-lyase (CSE), thereby modulating endogenous H_2_S production [23]. Based on this evidence, we propose a novel hypothesis: EE may exert neuroprotective effects by modulating the dopamine–H_2_S signaling axis, thereby influencing mitophagy and energy metabolism to ameliorate ischemia–reperfusion injury. This is the first study to systematically propose a functional association among EE, H_2_S signaling, and mitophagy. Our findings aim to elucidate the underlying mechanisms of non-pharmacological interventions in stroke rehabilitation and may offer new therapeutic targets and strategies for the clinical management of ischemic stroke.

## 2. Materials and Methods

### 2.1. Experimental Animals

Sexually mature male *C57BL/6* mice (aged 8–12 weeks, weighing 24–28 g) were procured from Zhejiang Vital River Laboratory Animal Technology Co., Ltd. (Jiaxing, China). This study adhered strictly to the guidelines for the Care and Use of Laboratory Animals published by the National Institutes of Health (NIH). All experimental protocols were reviewed and approved by the Experimental Animal Ethics Review Committee of Fudan University (Ethics approval number: 2020-Huashan Hospital-JS-163).

### 2.2. Antibody and Drug Administration

NaHS (207683-19-0; Sigma-Aldrich, St. Louis, MO, USA) and Mdivi-1 (S7162; Selleck Chemicals, Houston, TX, USA) were utilized for in vitro cellular experiments. YC-1 (2 mg/kg; B7641, APExBIO, Houston, TX, USA) was dissolved in 1% dimethyl sulfoxide (DMSO; 67-68-5; Sigma-Aldrich, St. Louis, MO, USA). Inhibitor groups the drug administration via the tail vein 12 h prior to cerebral ischemia model induction, followed by daily tail vein injections postoperatively to sustain low hypoxia-inducible factor 1-alpha (HIF-1α) expression [24]. Aminooxyacetic acid (AOAA; C13408; Sigma-Aldrich, St. Louis, MO, USA), an inhibitor targeting H_2_S-synthesizing enzymes cystathionine-β-synthase (CBS) and cystathionine-γ-lyase (CSE), was administered at 20 mg/kg (equivalent solvent as control) at two weeks and one week pre-surgery, with continued daily dosing postoperatively to maintain enzymatic suppression [25]. Primary antibodies used are detailed in Supplementary Table S1.

### 2.3. Animal Model Establishment

**Figure 1:**
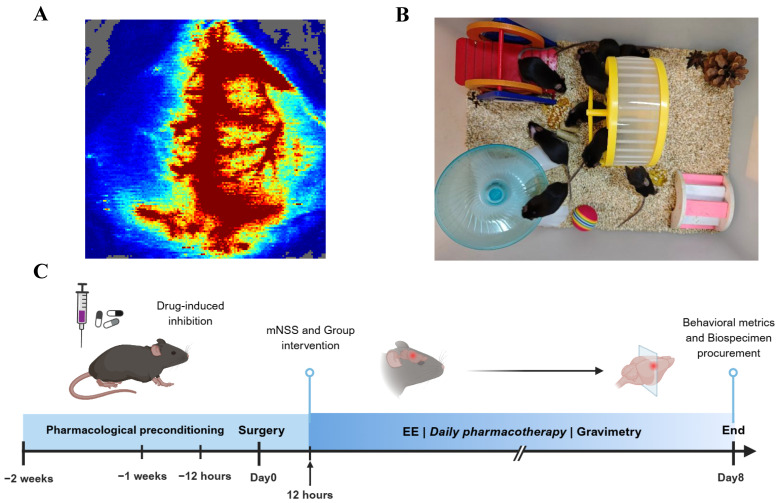
Schematic representation of animal experimental design. (**A**) Cerebral blood flow changes visualized by color Doppler ultrasonography during middle cerebral artery occlusion (MCAO) model induction in mice. (**B**) Apparatus configuration of the enriched environment (EE). (**C**) Experimental timeline schematic illustrating the procedural sequence of animal experiments.

Mice were initially anesthetized with 5% isoflurane for induction, followed by maintenance under 2% isoflurane continuous anesthesia. After preparing the surgical site through shaving and disinfection, a midline cervical incision was made to carefully expose the left neck muscles, fascia, and other soft tissues. The external carotid artery was ligated, while the common carotid artery and internal carotid artery were temporarily occluded using arterial clips. Subsequently, a 0.20 mm-diameter silicone-tipped nylon monofilament (Model L2000, Guangzhou Jialing Biotechnology Co., Ltd., Guangzhou, China) was inserted via an incision in the external carotid artery to occlude the origin of the middle cerebral artery. After one hour of occlusion, the filament was gently withdrawn, and the incision was sutured. Mice were then transferred to a thermostatic heating pad until full recovery from anesthesia. In the sham-operated group, all procedures were identical except for the insertion of the monofilament. Cerebral blood flow (rCBF) changes in all middle cerebral artery occlusion (MCAO) animals were monitored using a MoorLAB Laser Doppler Perfusion Monitor (Moor Instruments, Axminster, UK). Animals that died during the procedure or exhibited less than an 80% reduction in rCBF in the affected hemisphere compared to pre-ischemic levels were excluded from further analysis (Figure 1A).

### 2.4. Cell Culture and Oxygen-Glucose Deprivation/Reperfusion (OGD/R) Modeling

The SH-SY5Y neuroblastoma cell line (China Center for Type Culture Collection, CCTCC, Wuhan, China) was maintained in complete culture medium composed of MEM basal medium (Gibco 11-140-050, Grand Island, NY, USA) and Ham’s F12 nutrient mixture (Gibco 21-700-075, USA) in equal volumetric proportion. The medium was supplemented with 10% fetal bovine serum (FBS, Gibco 10-100-147) as growth factors and 1% penicillin-streptomycin antibiotic cocktail (Thermo Fisher Scientific, Waltham, MA, USA). Cell cultures were incubated in tri-gas incubators (Thermo Fisher Scientific, Waltham, MA, USA) under standard conditions (37 °C, 5% CO_2_, 95% humidity).

To establish the oxygen-glucose deprivation/reperfusion (OGD/R) model [26]. SH-SY5Y cells were cultured in glucose- and fetal bovine serum-free Hank’s Balanced Salt Solution (HBSS) within a hypoxic chamber (MIC-101; Billups-Rothenberg, Del Mar, CA, USA). A gas mixture of 95% N_2_ and 5% CO_2_ was continuously infused to maintain an oxygen concentration below 0.2%, which was dynamically monitored throughout the 4 h hypoxic incubation period to induce in vitro ischemic injury. After OGD treatment, cells were returned to normoxic conditions in complete culture medium and allowed to reoxygenate for 24 h.

NaHS (207683-19-0; Sigma-Aldrich, St. Louis, MO, USA) and/or Mdivi-1 (S7162; Selleck Chemicals, Houston, TX, USA), serving as an exogenous H_2_S donor and/or mitophagy inhibitor, respectively, were administered to designated treatment groups at 30 min pre-OGD/R exposure. Final concentrations were 200 μmol/L for NaHS and 25 μM for Mdivi-1 [27,28]. In addition, to activate dopamine signaling, a dopamine D1 receptor agonist, SKF38393 (5 μM; Sigma-Aldrich, St. Louis, MO, USA), was administered 30 min prior to OGD induction based on previously established protocols [29].

### 2.5. Cell Viability Assay

Cell viability was quantitatively analyzed using the Cell Counting Kit 8 (Absin, Shanghai, China). Absorbance was measured at 450 nm using a SpectraMax M5 microplate reader (Molecular Devices, San Jose, CA, USA). The cell viability percentage was calculated as follows: Cell viability (%) = [OD (test sample) − OD (blank control)]/[OD (control sample) − OD (blank control)] × 100.

### 2.6. Assessment of Infarction Volume

According to the classical experimental protocol, this study employed the 2,3,5-triphenyltetrazolium chloride (TTC) staining method (Sigma-Aldrich, St. Louis, MO, USA) to quantitatively assess the volume of cerebral infarction. The detailed operational procedure was as follows: Experimental animals were deeply anesthetized and subsequently decapitated. The entire brain tissue was promptly excised and coronal brain slices of uniform 2 mm thickness were then continuously prepared using a brain slice mold. These slices were immersed in a pre-heated 2% TTC staining solution at 37 °C and incubated for 30 min in the dark. This staining technique enabled color differentiation between the ischemic necrosis area (pale) and normal tissue (dark red) based on dehydrogenase activity levels. Subsequently, the NIH ImageJ image analysis system (version 1.53; NIH, Bethesda, MD, USA) was utilized for digital image acquisition and morphometric analysis of the stained brain slices. To eliminate the potential interference of cerebral edema on measurement outcomes, the infarct volume was calculated using the following formula: relative infarct ratio (%) = [(volume of healthy hemisphere) − (volume of normal area on affected side)]/(volume of healthy hemisphere) × 100%.

### 2.7. Western Blotting

Biological specimens were pretreated using a multi-fractionation lysis system in accordance with standardized protocols. Freshly harvested peri-infarct brain tissues and cultured cells were immediately homogenized in pre-chilled complete protein lysis buffer (Protein Extraction Kit, BB-3101, Bestbio, Shanghai, China) via mechanical disruption on ice. Mitochondrial protein fractions were isolated using a mitochondrial isolation kit (C3606, Beyotime, Shanghai, China) through differential centrifugation. Protein concentrations were quantified using the Biuret method and a BCA colorimetric assay kit (P0027, Beyotime, Shanghai, China), then adjusted to uniform concentrations with loading buffer. Protein samples were separated using 10% or 12% sodium dodecyl sulfate-polyacrylamide gel electrophoresis (SDS-PAGE) and transferred to 0.45 μm polyvinylidene difluoride (PVDF) membranes via wet transfer. Membranes were blocked with 5% non-fat milk in Tris-buffered saline with Tween-20 (TBST) at room temperature for 1 h. After three washes with TBST containing 0.1% Tween-20, membranes were incubated overnight at 4 °C with primary antibodies (1:1000 dilution), followed by 1 h incubation at room temperature with horseradish peroxidase (HRP)-conjugated anti-mouse (33201ES) or anti-rabbit (33101ES) secondary antibodies (Yeasen, Shanghai, China; 1:10,000 dilution). Signals were detected using an ECL Detection Reagent kit (36208ES, Yeasen, Shanghai, China) with a ChemiDoc Imaging System (Bio-Rad, Hercules, CA, USA) and quantified using ImageJ software (NIH, Bethesda, MD, USA).

### 2.8. Immunofluorescence

One week post-intervention, mice underwent transcardiac perfusion with pre-chilled saline followed by brain harvest. Brains were immediately fixed in 4% paraformaldehyde (4 °C, 24 h), then dehydrated through an ethanol gradient, paraffin-embedded, and sectioned (4 μm). Tissue sections were deparaffinized with xylene and rehydrated through an ethanol gradient to distilled water. Antigen retrieval was performed using EDTA-citrate buffer (pH 6.0). Endogenous peroxidases were blocked with 3% hydrogen peroxide, followed by membrane permeabilization with 0.5% Triton X-100 in PBS. After three PBS washes, sections were blocked with 3% goat serum in PBS (room temperature, 1 h). Primary antibodies were incubated overnight at 4 °C, followed by light-protected secondary antibody incubation (1 h, room temperature). Nuclei were counterstained with DAPI before mounting. For SH-SY5Y cells, medium was aspirated followed by PBS washes. Cells were fixed with 4% paraformaldehyde (15 min), permeabilized with methanol (10 min, −20 °C), and processed identically through blocking, antibody incubation, and mounting. Fluorescence distribution patterns and relative intensity quantification were analyzed using a FluoView FV3000 confocal microscope (Olympus Corporation, Tokyo, Japan).

### 2.9. Nissl Staining

After complete deparaffinization, brain paraffin sections were immersed in toluidine blue solution (Solarbio, Beijing, China) for 30 min staining at room temperature. Sections were thoroughly rinsed with running distilled water and differentiated in 95% ethanol until Nissl bodies exhibited dark blue staining against a pale blue background. Staining patterns were documented using a SlideView VS200 slide scanning system (Olympus Corporation, Tokyo, Japan).

### 2.10. Assessment of Neuronal Apoptosis

The Click-iT™ Plus TUNEL Assay (C10618; Thermo Fisher Scientific, USA) was employed to detect apoptotic neurons in the peri-infarct region of mouse brain tissue. Following the manufacturer’s protocol, deparaffinized and rehydrated sections were sequentially incubated with TdT Reaction Buffer, TdT Reaction Mixture, and Click-iT™ Plus Reaction Mix. Sections were then blocked with 3% goat serum in PBS (room temperature, 1 h) and incubated overnight with NeuN antibody (94403; Cell Signaling Technology, Danvers, MA, USA). Subsequent steps aligned with the immunofluorescence staining protocol. TUNEL-positive cells were quantified using a laser confocal microscope.

### 2.11. Transmission Electron Microscopy

The following modified preparation protocol was implemented for ultrastructural analysis: Tissue samples were precisely sectioned into 1–3 mm^3^ blocks using a sharp razor blade and immediately immersed in pre-chilled 2.5% glutaraldehyde (0.1 M phosphate buffer, pH 7.4) for primary fixation (≥3 h at 4 °C). After three 15 min rinses in 0.1 M phosphate buffer, secondary fixation was performed with 0.5% osmium tetroxide. Specimens underwent sequential processing including buffer rinses, progressive ethanol dehydration (50%, 70%, 90%, 100%), propylene oxide transition, and prolonged infiltration with Spurr resin. Polymerized blocks were sectioned into 70 nm slices using a Leica UC7 ultramicrotome (Leica Microsystems, Vienna, Austria). Sections were double-stained with 2% uranyl acetate in methanol (30 min, dark conditions) and Reynolds’ lead citrate (10 min). Ultrastructural observation and image acquisition were performed using a Talos 120C transmission electron microscope (Thermo Fisher Scientific, Eindhoven, The Netherlands) operated at 120 kV.

### 2.12. Quantification of MDA, T-AOC, and GSH

Following one week of respective treatments, serum samples were collected and fresh brain tissues were harvested via transcardiac perfusion from experimental mice. Peri-infarct brain tissues were homogenized in ice-cold buffer and centrifuged at 15,000× *g* for 15 min at 4 °C to obtain supernatant for oxidative stress analysis. Malondialdehyde (MDA) levels were quantified using a thiobarbituric acid (TBA) method-based assay kit [30] (A003-1-2; Nanjing Jiancheng Bioengineering Institute, Nanjing, China), total antioxidant capacity (T-AOC) was measured via ABTS method [31] (S0119, Beyotime, Shanghai, China), and total glutathione concentrations were determined using GSH and GSSG Assay Kit (S0053; Beyotime, China), strictly adhering to the manufacturers’ protocols for both brain tissue lysates and serum samples.

### 2.13. Mitochondrial Membrane Potential Assay

Mitochondrial membrane potential in SH-SY5Y cells was assessed using the JC-1 Assay Kit (T3168, Invitrogen, Carlsbad, CA, USA). Following cell culture in 6-well plates at appropriate confluency, medium was aspirated and cells were gently washed with PBS. Cells were incubated with JC-1 (10 μg/mL) at 37 °C for 15 min, protected from light. After three PBS washes to remove residual dye, fluorescence imaging was performed using an Olympus CKX53 inverted fluorescence microscope (Olympus, Japan), with red/green fluorescence ratios quantified to evaluate mitochondrial depolarization.

### 2.14. Mitochondrial Viability Assessment

According to a previous study [32] and the manufacturer’s instructions, SH-SY5Y cells grown on glass coverslips were washed with PBS to remove residual medium after respective treatments. MitoTracker Red CMXRos (C1035, Beyotime, Shanghai, China) was diluted in PBS to a final concentration of 50 nM. Cells were incubated with the dye at 37 °C for 30 min under light-protected conditions. Following dye removal, cells underwent fixation/permeabilization and subsequent immune-fluorescence staining with DAPI counterstaining or were directly mounted with DAPI. Mitochondrial fluorescence was quantified using a standard fluorescence microscope and an Olympus FluoView FV3000 confocal laser scanning microscope (Olympus Corporation, Tokyo, Japan).

ATP levels were measured using the Enhanced ATP Assay Kit based on firefly luciferase [33] (S0027, Beyotime, China). After respective treatments, cells were washed with pre-chilled PBS to remove residual medium and lysed in ATP lysis buffer. Following sufficient lysis, samples were centrifuged at 12,000× *g* for 5 min at 4 °C, and the supernatants were collected. Equal volumes of supernatant and ATP detection working solution were mixed and incubated, and luminescence was measured using a microplate reader (Molecular Devices, San Jose, CA, USA) to quantify ATP levels in each group.

### 2.15. Reactive Oxygen Species (ROS) Detection

Intracellular ROS levels were analyzed using the Reactive Oxygen Species Assay Kit (S0033, Beyotime, China). Briefly, the DCFH-DA fluorescent probe was diluted in PBS at a 1:1000 ratio. After removing culture medium from 6-well plates via PBS washes, cells from experimental groups were incubated with the diluted probe at 37 °C for 30 min under standard culture conditions. Fluorescence intensity, reflecting intracellular ROS levels, was quantified using an inverted fluorescence microscope (Olympus Corporation, Tokyo, Japan) with matched excitation/emission filters (488/525 nm).

### 2.16. Hydrogen Sulfide (H_2_S) Quantification

H_2_S levels in peri-infarct brain tissue, serum, and SH-SY5Y cells were quantified using the H_2_S-specific fluorescent probes HSip-1 and HSip-1 DA, following established methodology [34]. Fresh brain tissues were homogenized in PBS, and total protein concentrations were normalized using a BCA assay. Tissue homogenates and serum samples were incubated with 10 μM HSip-1 in deoxygenated PBS (pH 7.4) for 20 min at room temperature after centrifugation. Fluorescence intensity was measured using a Multiskan™ FC microplate reader (1410101; Thermo Fisher Scientific, USA). For cellular H_2_S detection, SH-SY5Y cells were incubated with 10 μM HSip-1 DA in deoxygenated PBS for 20 min at room temperature, followed by three PBS washes. Fluorescent images were acquired under standardized parameters using a fluorescence microscope (Olympus, Japan).

### 2.17. Body Weight Monitoring

Daily body weight measurements were recorded preoperatively and daily postoperatively using a precision electronic balance (±0.01 g resolution) to evaluate systemic functional status across experimental groups.

### 2.18. Neurobehavioral Assessment

The Modified Neurological Severity Score (mNSS) was conducted at postoperative 12 h and 1-week post-intervention. Quantitative neuromotor function was assessed at 1-week post-intervention through three standardized paradigms: Gait Analysis, Exploratory Behavior and Motor Coordination.

#### 2.18.1. mNSS

Neurological deficits were assessed at postoperative 12 h and 1-week post-intervention (prior to euthanasia) using an 18-point mNSS system. Two blinded investigators evaluated sensorimotor function across four domains: motor skills (limb symmetry/spontaneous activity), sensory responses (tactile/proprioceptive), balance coordination (beam walking), and reflexes (pinna/corneal/startle). Higher scores indicate more severe neurological impairment [35].

#### 2.18.2. CatWalk XT Gait Analysis

Gait function was quantified at 1-week post-intervention using the CatWalk XT system (Noldus, Wageningen, The Netherlands) with a 150 cm glass walkway. Paw contact intensity/duration and stride parameters (base of support, swing speed) were captured at 100 Hz. Three consecutive uninterrupted runs per mouse were analyzed, excluding trials with pauses >2 sec. Digital footprints were processed with CatWalk XT 10.6 software (version 10.6; Noldus, Wageningen, The Netherlands).

#### 2.18.3. Open Field Test

Mice were placed in the center of a 40 × 40 × 60 cm^3^ arena and allowed to acclimate for 5 min prior to automated tracking. Locomotor trajectories, immobility episodes, and rearing behaviors were recorded during a 10 min test session using video tracking software (SMART v3.0; Panlab, Barcelona, Spain). Quantitative parameters including total distance traveled, mean velocity (cm/s), and exploration behavior in the central area were analyzed for functional assessment.

#### 2.18.4. Rotarod Test

Preoperative acclimatization trials were conducted over three consecutive days using an accelerating rotarod (4–40 rpm progressive acceleration over 5 min). Mice failing to maintain balance for ≥200 s during the final training session were excluded. Post-intervention testing at 1-week recorded latency to fall (maximum 300 s), with trials terminated when mice exhibited passive rotation.

### 2.19. Co-Immunoprecipitation

Co-immunoprecipitation was performed to validate protein–protein interactions using the Immunoprecipitation Kit (ab206996, Abcam, Cambridge, UK). SH-SY5Y cells were trypsinized, washed with ice-cold PBS, and lysed in RIPA lysis buffer (20 mM Tris-HCl pH 7.4, 150 mM NaCl, 1% NP-40) supplemented with protease inhibitors on ice for 30 min. Cleared lysates were incubated overnight at 4 °C with specific primary antibodies: rabbit anti-Cystathionine γ-lyase (CSE; 12217-1-AP, Proteintech, Rosemont, IL, USA), rabbit anti-Calmodulin (CaM; 10541-1-AP, Proteintech, USA), or species-matched IgG control. Following immunocomplex formation, 40 μL protein A/G agarose beads were added for 4 h incubation at 4 °C. After five sequential washes, bound proteins were eluted and denatured in Laemmli buffer at 95 °C for 5 min prior to Western blotting analysis.

### 2.20. Flow Cytometry

Neuronal apoptosis was analyzed using the Annexin V-FITC Apoptosis Detection Kit (C1062, Beyotime, Shanghai, China) with flow cytometry. Following experimental treatments, cells were trypsinized, neutralized with complete culture medium, and centrifuged at 1,000 rpm for 10 min. Equal cell counts were resuspended in 195 μL Annexin V binding buffer and stained with 5 μL Annexin V-FITC plus 10 μL propidium iodide (PI) in the dark at 25 °C for 20 min. Fluorescence signals were quantified using a BD FACSCalibur flow cytometer (BD Biosciences, San Diego, CA, USA) under standardized optical configurations.

### 2.21. Housing Conditions

The mice with successful MCAO model establishment were randomly assigned to the MCAO group, EE group, AOAA group and YC-1 group. On the second day, except for the MCAO group, all the other groups were placed in the EE (Figure 1B) after the mNSS score assessment (Figure 1C). A variety of sensory and social stimuli were provided to the mice, which were achieved by configuring different taste, visual and tactile stimuli for each group. These included various types of bedding, spices with different flavors, and novel toy facilities such as seesaws, turntables, small balls, tunnels, stairs and shelters. As previously described [9], the EE provided mice with a substantially larger activity space (80 cm × 60 cm × 40 cm) compared to the standard housing conditions, and accommodated approximately 12 conspecifics per cage. To ensure novelty, the types and positions of various facilities were changed at fixed times every day. In contrast, the control group was housed in standard cages with limited dimensions (27 cm × 22.5 cm × 18 cm), and each cage contained only 4–6 mice during the same period.

### 2.22. High-Throughput RNA Sequencing

Transcriptomic analysis of mouse cerebral tissues was performed following standardized protocols from Guangzhou Gene Denovo Biotechnology Co., Ltd. (Guangzhou, China). Total RNA was isolated using TRIzol™ Reagent (Invitrogen, Carlsbad, CA, USA) with quality verification by NanoDrop ND-1000 spectrophotometry (Thermo Fisher Scientific, Wilmington, DE, USA) and RNA integrity assessment using the Agilent 2100 Bioanalyzer system (Agilent Technologies, Santa Clara, CA, USA). Qualified RNA samples underwent library preparation with TruSeq™ Stranded mRNA Library Prep Kit (Illumina, San Diego, CA, USA), followed by 150 bp paired-end sequencing on an Illumina NovaSeq 6000 platform. Raw reads were preprocessed via Trimmomatic (v0.39) for quality filtering, then aligned to the murine reference genome (GRCm38) using HISAT2 (v2.2.1). Differentially expressed genes were defined as those meeting |fold change| ≥ 1.5 with *p* ≤ 0.05. Hierarchical clustering and pathway enrichment analyses were conducted using Cluster 3.0 and ClusterProfiler R package (v4.2.2), respectively, with three biological replicates ensuring analytical robustness.

### 2.23. Statistical Analysis

All continuous data that conformed to a normal distribution were expressed and described as mean ± standard deviation. Except for the analysis of differences in body weight at different time points and between different groups, as well as the results of the mNSS score, which were analyzed using two-way ANOVA and Tukey’s test for multiple comparisons among groups, other multiple comparisons among groups were conducted using one-way ANOVA and Tukey’s test. The Dunnett’s multiple comparison test was used for the results of Cell Viability. Statistical analyses were performed using SPSS Statistics v22.0 (IBM Corp., Armonk, NY, USA) and GraphPad Prism v8.0 (GraphPad Software, La Jolla, CA, USA). Statistical significance was defined as *p* < 0.05, with annotations as follows: ns (not significant), * *p* < 0.05, ** *p* < 0.01, *** *p* < 0.001, **** *p* < 0.0001.

## 3. Results

### 3.1. EE Exerts Neuroprotective Effects by Attenuating Neuronal Apoptosis and Reducing Infarct Volume After Cerebral Ischemia–Reperfusion Injury

**Figure 2:**
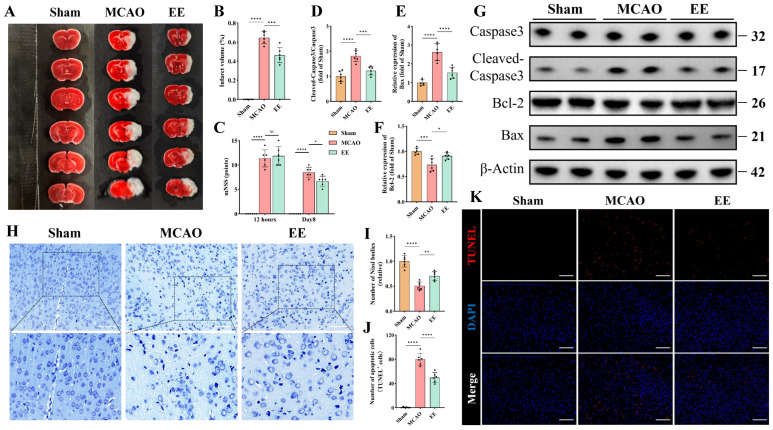
EE confers neuroprotective effects during the acute phase of cerebral ischemia–reperfusion injury. (**A**) The 2,3,5-triphenyltetrazolium chloride (TTC) staining illustrating infarct areas in the ischemic hemisphere. (**B**) Quantification of infarct volume (*n* = 6). (**C**) Statistical graph of neurological deficit scores in mice with cerebral ischemia on the first and eighth days after surgery (*n* = 6). (**D**–**G**) Representative Western blot bands of apoptosis-related markers and quantitative analysis of the expression levels of apoptosis-associated proteins (*n* = 6). (**H**) Representative Nissl staining images showing neuronal morphology and survival; scale bar: 100 μm. (**I**) Quantitative analysis of Nissl body density (*n* = 6). (**J**,**K**) Representative TUNEL staining images of the peri-infarct cortex and quantification of apoptotic cells; scale bar: 100 μm (*n* = 6). All data are presented as mean ± standard deviation (SD). Two-way ANOVA followed by Tukey’s post hoc test was used for multiple comparisons of the Modified Neurological Severity Score (mNSS) across different groups and time points. For all other intergroup comparisons, one-way ANOVA followed by Tukey’s post hoc test was applied. ns, not significant; * *p* < 0.05, ** *p* < 0.01, *** *p* < 0.001, **** *p* < 0.0001.

Cerebral infarct volumes were significantly increased in the MCAO group compared to Sham. EE intervention for one week markedly reduced infarct size versus the MCAO model group (*p* < 0.001, Figure 2A,B). The neurological deficit score test was used to evaluate the overall motor function of mice with cerebral ischemia. The mNSS score demonstrated comparable baseline neurological deficits between MCAO and EE groups. After one-week intervention, EE-treated mice exhibited significant functional recovery (*p* < 0.05, Figure 2C). Nissl bodies and Nissl staining were used to assess the morphology of neurons after cerebral ischemia. Nissl staining of peri-infarct neurons revealed severe neuronal loss, sparse cellular arrangements, atrophic nuclei, and diminished Nissl bodies in the MCAO group (*p* < 0.0001), with pronounced cytoplasmic vacuolation. EE treatment significantly restored Nissl body density (*p* < 0.01) and ameliorated neuronal morphology, rescuing neuronal functionality (Figure 2H,I). Western blotting was used to assess the expression levels of apoptosis-related factors. The results showed that compared to the MCAO group, the ratio of Cleaved Caspase3/Caspase3 and the expression of Bax were significantly inhibited by EE intervention as expected (*p* < 0.001; Figure 2D,E,G). EE significantly improved the expression of the antiapoptotic factor Bcl-2 (*p* < 0.05, Figure 2F,G). The changes in the expression levels of Bcl-2 and Bax (Figure 2E,F) were consistent with the previous research results of our group [9]. Because Bcl-2 and Bax are mainly located on the mitochondria and are closely related to mitochondrial membrane permeability and pores on the mitochondria [36]. Studies have shown that the Bcl-2 protein family is a key regulator of mitochondria-related apoptosis and can initiate or inhibit cell death [37]. Building upon our previous findings regarding the effects of EE on mitophagy [9], the dynamic alterations of mitochondria following cerebral ischemia and their implications for neurological function warrant further investigation. Consistent with this, TUNEL staining (Figure 2K) revealed that EE intervention significantly attenuated neuronal apoptosis compared to the ischemia group (*p* < 0.0001; Figure 2J).

### 3.2. EE Demonstrates Significant Regulatory Effects on Mitochondrial Dynamics and Mitophagy Following Cerebral Ischemia–Reperfusion Injury

**Figure 3:**
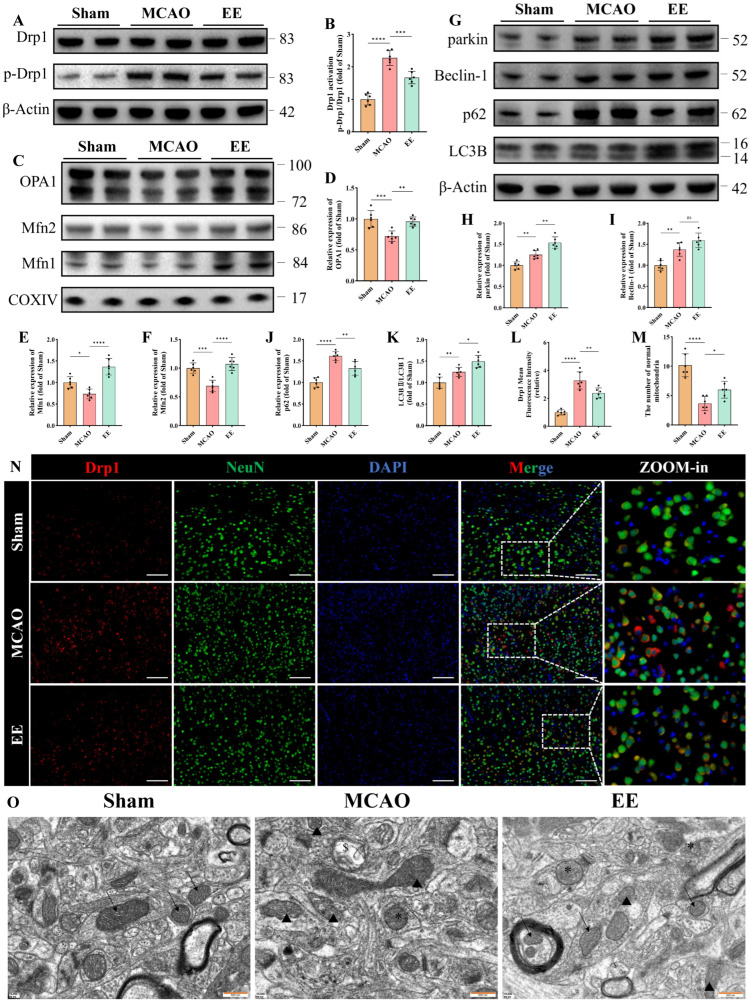
EE improves mitochondrial dynamics and mitophagy following cerebral ischemia–reperfusion injury. (**A**,**B**) Representative Western blot bands and quantitative analysis of the mitochondrial fission marker Drp1 (*n* = 6). (**C**–**F**) Representative Western blot images and statistical quantification of mitochondrial fusion markers OPA1, Mfn1, and Mfn2 (*n* = 6). (**G**–**K**) Representative Western blot images and quantitative analysis of mitophagy-related proteins, including parkin, Beclin-1, p62, and LC3B (*n* = 6). (**N**) Representative immunofluorescence results of tissue sections from peri-infarct brain tissue, showing the differential expression of Drp1 among the three groups, scale bar: 50 μm. (**L**) Quantitative analysis of Drp1 immunofluorescence intensity using ImageJ (*n* = 6). (**M**,**O**) Representative transmission electron microscopy (TEM) images of peri-infarct mitochondria and the number of normal mitochondria in each image: black arrows indicate healthy mitochondria (intact cristae and matrix); triangles denote damaged mitochondria (spherical swelling, fragmented cristae); “$” denotes lysosomal engulfment of mitochondria (single-membrane structures); “*” denotes autophagosomes containing mitochondria (double-membrane structures); scale bar: 500 nm (*n* = 6). All values are expressed as mean ± standard deviation (SD). One-way ANOVA followed by Tukey’s post hoc test was used for multiple group comparisons. ns, not significant; * *p* < 0.05, ** *p* < 0.01, *** *p* < 0.001, **** *p* < 0.0001.

Mitochondrial dynamics and mitophagy play crucial roles in mitochondrial quality control and functional maintenance. Western blot results demonstrated disrupted mitochondrial fission-fusion equilibrium following cerebral ischemia–reperfusion injury, with excessive mitochondrial fission observed in the MCAO group. This was evidenced by a significantly increased p-Drp1/Drp1 ratio compared to the Sham group (Figure 3A,B, *p* < 0.0001). Protein analysis of isolated mitochondria revealed significant downregulation of OPA1, Mfn2 and Mfn1 expressions in the MCAO group (Figure 3C–F, *p* < 0.05). Notably, EE treatment partially reversed the pathological elevation of p-Drp1/Drp1 ratio and restored the expression levels of mitochondrial fusion regulators Mfn1, Mfn2, and OPA1 in ischemic brains (Figure 3A–F). Collectively, these findings indicate that EE ameliorates mitochondrial dynamics in peri-infarct neurons of cerebral ischemic mice, which was further corroborated by Drp1 immunofluorescence results (Figure 3L,N, *p* < 0.01) and ultrastructural evidence from transmission electron microscopy (TEM) (Figure 3O). TEM analysis demonstrated distinct cristae structure and homogeneous matrix density in Sham group mitochondria (Figure 3O). In contrast, MCAO group mitochondria exhibited characteristic pathological alterations including spherical swelling, cristae fragmentation, discontinuous membrane integrity, along with typical mitochondrial fission and mitophagic features. Importantly, EE intervention attenuated mitochondrial damage and improved autophagic clearance efficiency (Figure 3M,O). These observations were consistent with the quantitative Western blot analysis results (Figure 3G). Although no statistically significant difference in Beclin-1 expression was observed between the treatment and model groups, EE exhibited a tendency to enhance its expression (Figure 3I). Notably, EE significantly increased the expression level of the mitophagy marker parkin (Figure 3H, *p* < 0.01), as well as the LC3B-II/LC3B-I ratio (Figure 3K, *p* < 0.05), compared with the ischemia group. Importantly, p62 accumulation was markedly reduced in the peri-infarct cortex of EE-treated mice (Figure 3J, *p* < 0.01), suggesting that the autophagic flux was not impaired and that autophagosomes were effectively degraded. Collectively, these results indicate that EE ameliorates mitochondrial dynamics and facilitates the mitophagy process in the peri-infarct region following cerebral ischemia.

### 3.3. EE Modulates Endogenous H_2_S Production, Mitigates Oxidative Stress, and Preserves Mitochondrial Function Post-CIRI

**Figure 4:**
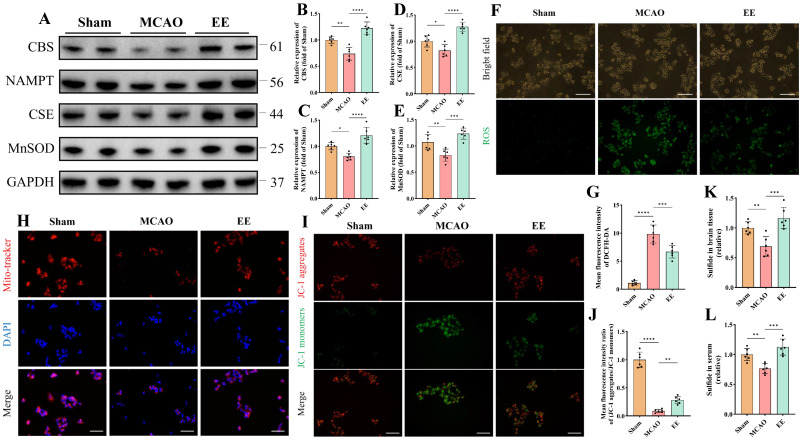
EE modulates endogenous H_2_S production, alleviates oxidative stress, and improves mitochondrial function following cerebral ischemia–reperfusion injury (CIRI). (**A**) Representative Western blot bands and normalized quantitative analyses of (**B**) CBS, (**C**) NAMPT, (**D**) CSE, and (**E**) MnSOD protein expressions in peri-infarct brain tissues, respectively (*n* = 6). (**F**) Representative immunofluorescence images of intracellular total oxidative stress levels detected using the DCFH-DA fluorescent probe; scale bar: 100 μm. (**G**) Quantification of mean DCFH-DA fluorescence intensity (*n* = 6). (**H**) Representative fluorescence images of mitochondrial morphology and distribution detected using the mitochondria-specific probe MitoTracker; scale bar: 50 μm (*n* = 6). (**I**) Representative fluorescence images of mitochondrial membrane potential assessed by the JC-1 assay; scale bar: 50 μm. (**J**) Quantitative analysis of mitochondrial membrane potential (*n* = 6). (**K**,**L**) H_2_S content in peri-infarct brain tissues and serum samples (*n* = 6). All values are expressed as mean ± standard deviation (SD). One-way ANOVA followed by Tukey’s post hoc test was used for multiple group comparisons. * *p* < 0.05, ** *p* < 0.01, *** *p* < 0.001, **** *p* < 0.0001.

Neuronal oxidative stress, primarily stemming from mitochondrial impairment and secondary cytokine storms, constitutes a critical pathological manifestation of cerebral ischemia–reperfusion injury (CIRI) [38]. Studies indicate that H_2_S may exert neuroprotective effects through antioxidative stress, anti-inflammatory responses, apoptosis downregulation, and autophagy modulation [39]. To investigate whether EE influences H_2_S expression and oxidative stress levels in ischemic brains, peri-infarct brain tissues were harvested for Western blot quantitative analysis, while cerebral tissues and serum samples were simultaneously collected for H_2_S concentration measurements. Protein quantification revealed significant reductions in key endogenous H_2_S-producing enzymes (CBS and CSE) and the antioxidative stress protein nicotinamide phosphoribosyltransferase (NAMPT) and manganese superoxide dismutase (MnSOD) in the MCAO group (*p* < 0.05, Figure 4A–E). These alterations were significantly reversed after one-week EE intervention (*p* < 0.001, Figure 4A–E). HSip-1 fluorescence probes demonstrated decreased H_2_S levels in MCAO peri-infarct tissues and serum compared to Sham controls, which were ameliorated by EE (Figure 4K,L). Subsequently, serum samples collected from different mice within the same group were individually added to the culture medium of SH-SY5Y cells 30 min prior to the induction of the OGD/R model. The serum was applied at a volume ratio of 1:4 (serum: medium) in each well. After 24 h of reoxygenation, the cells were subjected to assessments of reactive oxygen species (ROS) production and mitochondrial function (Figure 4F–J). DCFH-DA fluorescence microscopy showed significantly elevated intracellular ROS in MCAO versus Sham, while EE mitigated this effect (Figure 4F,G). Mitochondrial morphology and function evaluated via MitoTracker and JC-1 kits revealed weakened MitoTracker fluorescence intensity and perinuclear mitochondrial clustering in MCAO cells compared to Sham (Figure 4H,I), suggesting early-stage mitochondrial dysfunction that may trigger ROS overproduction and subsequent cell death [40]. JC-1 staining confirmed pronounced mitochondrial membrane potential collapse in MCAO, evidenced by reduced JC-1 aggregate/monomer ratios, with EE significantly restoring Mitochondrial membrane potential (Figure 4I,J). Consistent with previous findings [41], EE enhanced mitochondrial biogenesis. Western blot analysis of neurons treated with conditioned serum further indicated that EE may promote mitochondrial biogenesis through the SIRT1/PGC-1α pathway (Figure S7). This observation is in line with the MitoTracker staining results (Figure 4H).

### 3.4. H_2_S Ameliorates Mitophagy and Mitochondrial Function in Ischemic-Hypoxic Neurons

Dysfunctional mitochondria are selectively cleared via mitophagy, a critical mechanism in cerebral ischemia–reperfusion injury [42]. While the PINK1/parkin pathway dominates canonical mitophagy research, the HIF-1α/BNIP3L axis has been implicated only in renal ischemia–reperfusion models [43]. To investigate H_2_S-mediated neuroprotection against oxidative stress and mitophagy interplay, oxygen-glucose deprivation/reoxygenation (OGD/R)-treated neurons were co-cultured with the H_2_S donor NaHS (200 μmol/L) and mitophagy inhibitor Mdivi-1 (25 μM), based on prior pharmacological validations [27,28] and cytotoxicity assays (Figure S1).

**Figure 5:**
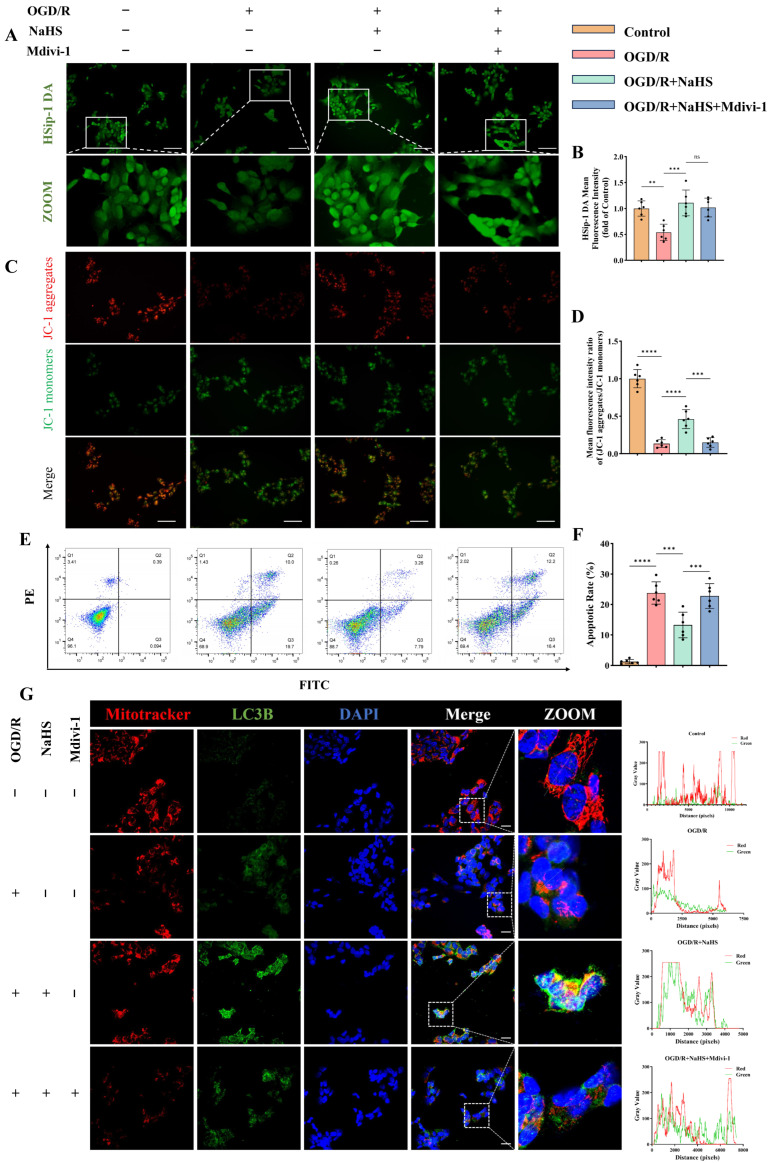
Effects of hydrogen sulfide (H_2_S) on mitophagy and apoptosis in neurons subjected to ischemic-hypoxic injury. (**A**) Representative fluorescence images of intracellular H_2_S levels in SH-SY5Y neurons detected using the H_2_S-specific fluorescent probe HSip-1 DA; scale bar: 50 μm. (**B**) Quantitative analysis of the mean fluorescence intensity of HSip-1 DA (*n* = 6). (**C**,**D**) Representative JC-1 fluorescence images assessing mitochondrial membrane potential and the corresponding quantitative analysis; scale bar: 50 μm (*n* = 6). (**E**,**F**) Representative flow cytometry plots and quantitative analysis of neuronal apoptosis across different groups (*n* = 6). (**G**) Representative immunofluorescence images of co-staining with the mitochondria-specific probe MitoTracker and the autophagy marker LC3B, along with fluorescence co-localization profiles along the white solid lines in each group; scale bar: 20 μm. All data are presented as mean ± standard deviation (SD). One-way ANOVA followed by Tukey’s post hoc test was used for multiple comparisons. ns, not significant; ** *p* < 0.01, *** *p* < 0.001, **** *p* < 0.0001.

Significantly reduced H_2_S levels were observed in OGD/R-treated neurons (*p* < 0.01, Figure 5A,B), while NaHS co-culture markedly increased HSip-1 DA fluorescence intensity (*p* < 0.001, Figure 5A,B). H_2_S content remained unaltered upon mitophagy inhibitor administration (*p* > 0.05, Figure 5A,B). JC-1 assays revealed mitochondrial depolarization through decreased aggregate-to-monomer ratios post-OGD/R (*p* < 0.0001, Figure 5C,D), indicative of functional impairment. H_2_S supplementation restored membrane potential (*p* < 0.0001, Figure 5C,D), whereas mitophagy inhibition exacerbated depolarization (*p* < 0.001, Figure 5C,D).

Mitochondrial morphology and distribution visualized using the mitochondria-specific probe MitoTracker also yielded consistent results (Figure 5G and Figure S2B). Flow cytometry analysis revealed the overall functional status of neurons. As shown, oxygen-glucose deprivation induced substantial neuronal death (Figure 5E). Notably, H_2_S supplementation significantly rescued neurons from OGD/R-induced apoptosis (*p* < 0.001; Figure 5E,F). As expected, inhibition of mitophagy further exacerbated neuronal dysfunction, resulting in a marked increase in apoptotic cells (*p* < 0.001; Figure 5E,F). Similar findings were observed in LC3B immunofluorescence staining (Figure 5G). H_2_S treatment markedly enhanced the mean fluorescence intensity of LC3B and Parkin, whereas this effect was attenuated in the presence of an autophagy inhibitor (Figure 5G and Figure S2B–F).

Co-localization analysis of MitoTracker and LC3B signals was performed using ImageJ software. As illustrated, H_2_S administration significantly promoted LC3B-mitochondria co-localization, while this enhancement was partially reversed upon autophagy inhibition (Figure 5G).

**Figure 6:**
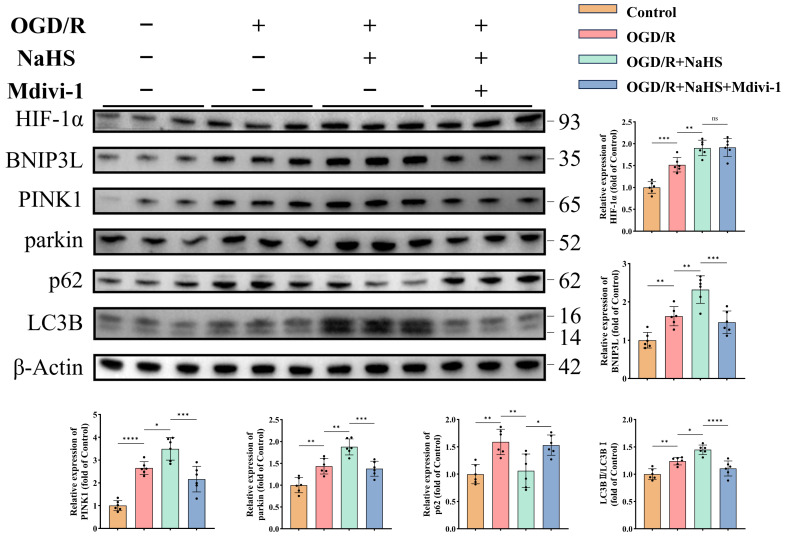
H_2_S enhances both canonical and non-canonical mitophagy pathways in neurons under ischemic-hypoxic conditions. Representative Western blot bands and quantitative analysis of normalized expression levels of mitophagy-related proteins (*n* = 6). All data are presented as mean ± standard deviation (SD). One-way ANOVA followed by Tukey’s post hoc test was used for multiple group comparisons. ns, not significant; * *p* < 0.05, ** *p* < 0.01, *** *p* < 0.001, **** *p* < 0.0001.

Western blot analysis revealed H_2_S-mediated activation of both canonical (PINK1/parkin) and non-canonical (HIF-1α/BNIP3L) mitophagy pathways (*p* < 0.05, Figure 6), with enhanced autophagic flux evidenced by increased LC3B-II/I ratios (*p* < 0.05, Figure 6) and reduced p62 accumulation (*p* < 0.01, Figure 6). Mitophagy inhibitor treatment suppressed autophagy-related protein expression and reversed p62 reduction (*p* < 0.05, Figure 6), while exerting no effect on HIF-1α (*p* > 0.05, Figure 6). These findings suggest that H_2_S ameliorates both canonical and non-canonical mitophagy pathways in neurons subjected to ischemic-hypoxic injury, which is critical for preserving neuronal and mitochondrial function.

Moreover, DCFH-DA staining indicated that H_2_S effectively attenuated the OGD/R-induced increase in oxidative stress levels in neurons, an effect that was reversed by mitophagy inhibition (Figure S2A,C).

Collectively, these results indicate that H_2_S may suppress oxidative stress, improve mitochondrial morphology and function, and ultimately preserve neuronal integrity and inhibit apoptosis by enhancing the mitophagy process under ischemic-hypoxic conditions.

### 3.5. EE Ameliorates Mitophagy and Mitochondrial Function via H_2_S Regulation

**Figure 7:**
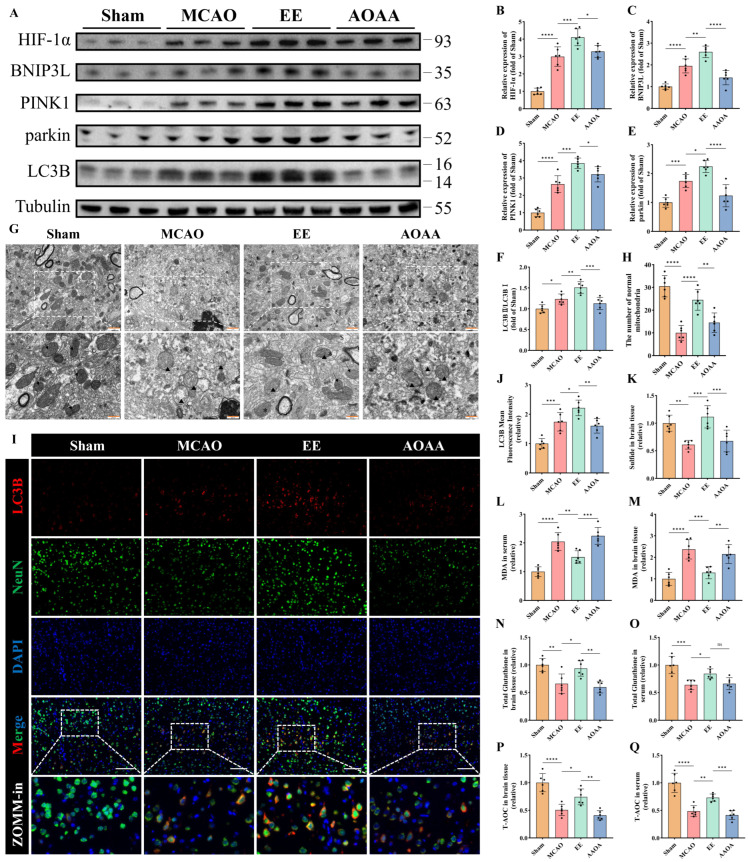
EE improves mitophagy and mitochondrial function by modulating endogenous H_2_S production. (**A**) Representative Western blot bands of mitophagy markers in peri-infarct brain tissues. (**B**–**F**) Quantitative analysis of normalized expression levels of HIF-1α, BNIP3L, PINK1, parkin, and the LC3B-II/LC3B-I ratio (*n* = 6). (**G**) TEM images of peri-infarct mitochondria: black arrows indicate healthy mitochondria (intact cristae and matrix); triangles denote damaged mitochondria (spherical swelling, fragmented cristae); “$” indicates single-membrane mitolysosomes; “*” indicates double-membrane mitophagosomes. Scale bars: 1 μm and 500 nm. (**H**) The number of normal mitochondria corresponding to each field of view in the TEM images (*n* = 6). (**I**,**J**) Representative immunofluorescence images of the mitophagy marker LC3B in brain tissue sections and quantification of mean fluorescence intensity; scale bar: 100 μm (*n* = 6). (**K**) Relative H_2_S levels in peri-infarct tissues (*n* = 6). (**L**–**Q**) Quantitative analyses of malondialdehyde (MDA), total glutathione, and total antioxidant capacity (T-AOC) in peri-infarct brain tissue and serum samples (*n* = 6). All values are presented as mean ± standard deviation (SD). One-way ANOVA followed by Tukey’s post hoc test was used for multiple comparisons. ns, not significant; * *p* < 0.05, ** *p* < 0.01, *** *p* < 0.001, **** *p* < 0.0001.

MCAO mice and aminooxyacetic acid (AOAA) [25], an inhibitor of endogenous H_2_S-synthesizing enzymes cystathionine β-synthase/cystathionine γ-lyase (CBS/CSE), were utilized. Western blot analysis demonstrated that EE significantly enhanced mitophagy in peri-infarct brain tissues, potentially mediated through PINK1/parkin and HIF-1α/BNIP3L pathways (*p* < 0.05, Figure 7A–F). These effects were abolished upon H_2_S synthesis inhibition (*p* < 0.05, Figure 7A–F). Transmission electron microscopy revealed EE alleviated mitochondrial swelling, preserved cristae integrity, and increased mitophagic vacuoles and the number of normal mitochondria compared to MCAO controls, all reversed by AOAA (Figure 7G,H). Immunofluorescence confirmed EE-induced LC3B upregulation in peri-infarct regions, suppressed by AOAA (*p* < 0.05, Figure 7I,J). Quantitative H_2_S assays validated EE-mediated H_2_S restoration and AOAA efficacy (Figure 7K). It is reasonable to speculate that the neuroprotective effects of EE may be mediated, at least in part, through the regulation of endogenous H_2_S production in mice following cerebral ischemia.

Quantitative analyses of oxidative stress markers in both peri-infarct brain tissue and serum revealed that EE significantly improved the levels of malondialdehyde (MDA) (*p* < 0.01 and *p* < 0.001, Figure 7L,M), total glutathione (*p* < 0.05, Figure 7N,O), and total antioxidant capacity (T-AOC) (*p* < 0.05 and *p* < 0.01, Figure 7P,Q) in MCAO mice. However, when endogenous H_2_S synthesis was inhibited, the beneficial effects of EE on MDA, total glutathione, and T-AOC were markedly diminished (Figure 7L–Q). Interestingly, there were no significant differences in the expression of NAMPT and MnSOD in the peri-infarct cortex between the EE treatment group and the AOAA (H_2_S synthesis inhibitor) group (*p* > 0.05, Figure S3).

Taken together, these in vivo and in vitro findings suggest that EE may enhance mitophagy and mitochondrial function by modulating endogenous H_2_S production, thereby attenuating oxidative stress and improving overall neuronal viability following cerebral ischemia. Although EE also upregulated the expression of NAMPT and MnSOD, the relationship between H_2_S and these factors remains unclear, indicating that H_2_S may not exert its protective effects by directly promoting their expression.

### 3.6. EE Activates the Non-Canonical HIF-1α/BNIP3L/LC3B Mitophagy Pathway via H_2_S Modulation

**Figure 8:**
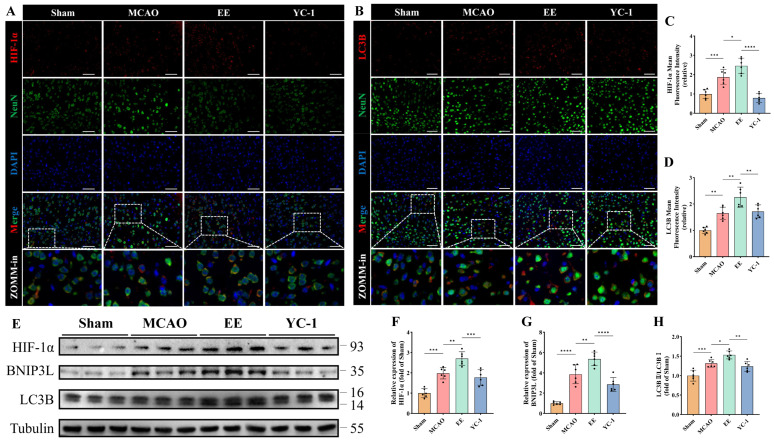
EE modulates the non-canonical mitophagy pathway via regulation of H_2_S production. (**A**,**C**) Representative double immunofluorescence images showing co-localization of HIF-1α and NeuN in brain tissue sections, and quantitative analysis of mean HIF-1α fluorescence intensity; scale bar: 50 μm (*n* = 6). (**B**,**D**) Representative double immunofluorescence images showing co-localization of LC3B and NeuN, along with quantitative analysis of LC3B expression; scale bar: 50 μm (*n* = 6). (**E**) Western blot image of mitophagy-related proteins in peri-infarct tissues. (**F**–**H**) Quantitative analysis of HIF-1α, BNIP3L, and the LC3B-II/LC3B-I ratio, respectively (*n* = 6). All data are presented as mean ± standard deviation (SD). One-way ANOVA followed by Tukey’s post hoc test was used for multiple group comparisons. * *p* < 0.05, ** *p* < 0.01, *** *p* < 0.001, **** *p* < 0.0001.

To further investigate the mechanism underlying mitophagy, the involvement of the non-canonical HIF-1α/BNIP3L/LC3B pathway was specifically examined and validated using YC-1 (Selleck Chemicals, Houston, TX, USA), a pharmacological inhibitor of HIF-1α stabilization [24]. Quantitative analysis results of protein demonstrated that EE significantly enhanced mitophagy activation through this pathway (*p* < 0.05, Figure 8E–H), with this enhancement being markedly attenuated by HIF-1α inhibition (*p* < 0.01, Figure 8E–H). Immunofluorescence imaging confirmed EE-mediated upregulation of HIF-1α (*p* < 0.05, Figure 8A,C) and LC3B (*p* < 0.01, Figure 8B,D) in peri-infarct regions. Consistent with this, results from the YC-1 treatment group indicated that inhibition of HIF-1α led to a significant reduction in the expression of the autophagy marker LC3B (*p* < 0.05; Figure 8B,D), functionally linking HIF-1α signaling to autophagic flux modulation.

### 3.7. EE-Mediated H_2_S Regulation Improves Neuronal Function via Mitophagy Restoration in Cerebral Ischemia

**Figure 9:**
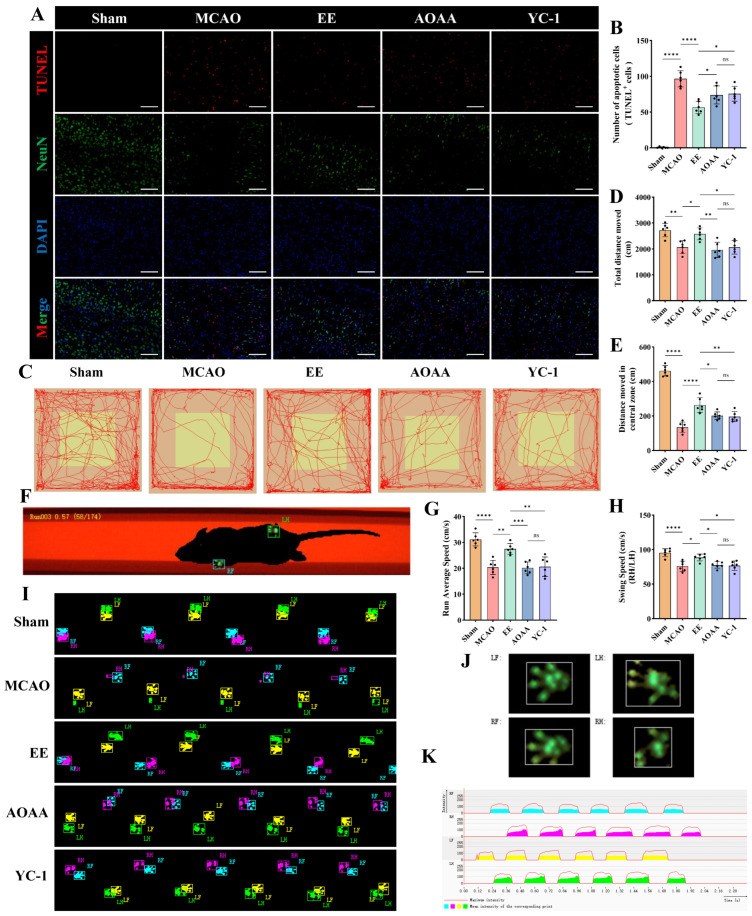
EE improves neurological function in mice following cerebral ischemia. (**A**,**B**) Representative TUNEL staining of peri-infarct brain tissue sections and corresponding quantitative analysis; scale bar: 100 μm (*n* = 6). (**C**) Open field locomotor trajectories illustrating exploratory patterns. (**D**,**E**) Total locomotion distance and central zone exploration in open field (*n* = 6). (**F**) Schematic diagram of mice undergoing the CatWalk gait analysis system. (**G**,**H**) Average locomotor velocity and the ratio of swing speed between the right hindlimb (RH) and left hindlimb (LH) in each group (*n* = 6). (**I**) Representative paw print images (PrintView) from the CatWalk analysis. (**J**) Representative hemiplegic-limb footprint patterns and (**K**) Real-time PrintIntensities heatmaps from MCAO mice. All data are presented as mean ± standard deviation (SD). One-way ANOVA followed by Tukey’s post hoc test was used for multiple group comparisons. ns, not significant; * *p* < 0.05, ** *p* < 0.01, *** *p* < 0.001, **** *p* < 0.0001.

To assess the neuroprotective effects of EE in ischemic mice, histological staining and behavioral evaluations were conducted before sacrifice in each group. Dual TUNEL/NeuN immunofluorescence demonstrated extensive neuronal death in MCAO peri-infarct zones, which was markedly rescued by EE (*p* < 0.0001, Figure 9A,B). H_2_S or HIF-1α inhibition partially attenuated this neuroprotection (*p* < 0.05, Figure 9A,B). Overall, the body weight and Rotarod test performance of mice with cerebral ischemia–reperfusion injury showed significant improvement after intervention for a period of time (Figure S4). Specifically, starting from the 6th day after surgery, the body weight of the treatment group mice showed a significant difference compared with the model group (*p* < 0.05, Figure S4A). Notably, when endogenous H_2_S was inhibited, the body weight of the mice began to show a difference on the 5th day (*p* < 0.05, Figure S4A). After one week of treatment, the time that the cerebral ischemia mice could stay on the rod was significantly prolonged (*p* < 0.01, Figure S4B). These may indicate the long-term protective effect of EE. In contrast, the overall improvement effect of EE was significantly inhibited by AOAA and YC-1 (Figure S4A,B). Subsequently, the open field test was conducted to evaluate the overall locomotor activity and exploratory behavior of the mice. Compared with the sham group, mice in the MCAO group exhibited a marked reduction in both total travel distance and distance traveled within the central zone, with a particularly pronounced decline in central zone exploration (*p* < 0.01, Figure 9C–E). EE intervention significantly improved both general locomotor performance and exploratory behavior in ischemic mice (*p* < 0.05, Figure 9C–E), with a notably enhanced central zone activity (*p* < 0.0001, Figure 9C,E). Notably, when the contents of H_2_S and HIF-1α were reduced, the neuroprotective effect of EE intervention might be significantly reduced (*p* < 0.05, Figure 9C–E). Then, the Catwalk test was conducted on the mice in each group, and the schematic diagram and representative footprint images are shown (Figure 9F,I). The representative gait results of the MCAO group mice were characterized by a significant reduction in the contact area of the affected side, especially the right hind limb (Figure 9J), a decrease in the plantar pressure of the affected side, and an extension of the foot clearance (support phase) time (Figure 9K). EE significantly improved the overall movement speed and the ratio of swing phase speed of right hindlimb (RH)/left hindlimb (LH) in the cerebral ischemia mice (*p* < 0.05, Figure 9G, H), that is, both the overall movement speed and the swing speed of the affected hind limb were significantly improved. However, these improvement effects were reversed by the inhibitors of H_2_S and HIF-1α (*p* < 0.05, Figure 9G,H).

### 3.8. EE Potentiates H_2_S Biosynthesis via Calmodulin Signaling Orchestrated by Dopamine Modulation

**Figure 10:**
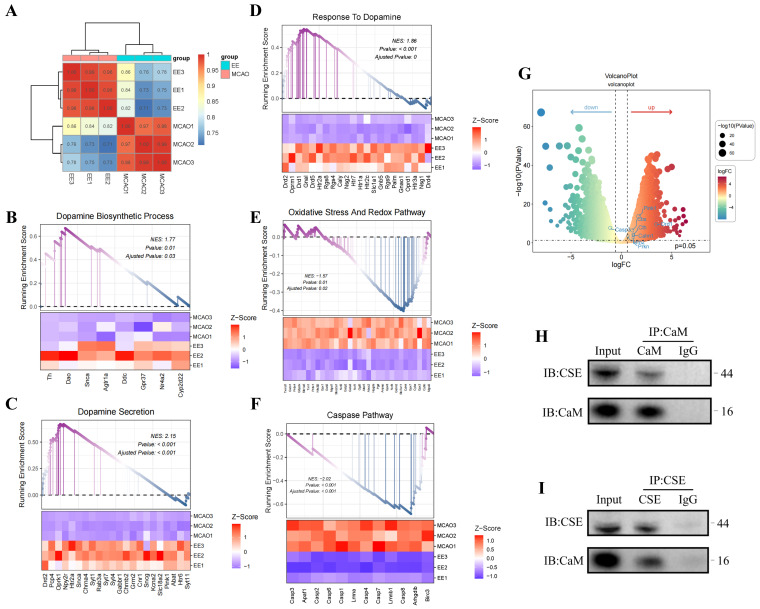
Potential mechanism by which EE regulates endogenous H_2_S biosynthesis. (**A**) Sample cluster analysis of transcriptomic sequencing. (**B**–**F**) Gene Set Enrichment Analysis (GSEA) plots for dopamine biosynthetic process, dopamine secretion, response to dopamine, oxidative stress pathway, and caspase pathway in mouse cerebral tissues (*n* = 3). (**G**) Volcano plot demonstrating upregulated expression of calmodulin-associated gene *Calm1*, H_2_S-synthesizing enzyme genes *Cbs* and *Cth*, dopamine receptor gene *Drd1*, along with enhanced expression of mitophagy-related genes *Pink1* and *Prkn* (encoding parkin), and mitochondrial fusion gene *Mfn2* in the EE group versus the model group. Conversely, EE intervention markedly suppressed apoptosis-related gene *Casp3* expression (*n* = 3, |log_2_FC| > 1.5, *p* < 0.05). (**H**,**I**) Representative co-immunoprecipitation (Co-IP) blot images.

To delineate mechanistic basis of EE for H_2_S regulation, transcriptomic sequencing and bioinformatics analyses were employed. Cluster analysis of cerebral transcriptomes revealed distinct intergroup segregation (Figure 10A). Gene Set Enrichment Analysis (GSEA) demonstrated EE-driven upregulation of dopamine biosynthetic processes (GO:0042417), dopamine secretion (GO:0014046), and dopamine response pathways (Figure 10B–D). These findings align with prior evidence [29] implicating dopamine receptor 1 (DR1)-CSE/H_2_S axis activation in calmodulin (CaM)-mediated CSE stimulation. Therapeutic upregulation of monoamine GPCR signaling (KEGG:04020), dopamine receptor binding (GO:0035240), and D1 receptor binding (GO:0031876) further corroborated this mechanism (Figure S5). Co-immunoprecipitation confirmed CaM-CSE molecular interactions in neurons (Figure 10H,I), suggesting EE enhances dopamine synthesis/secretion to regulate neuronal Ca^2+^ flux and CaM expression, thereby activating H_2_S-synthetic enzymes. GSEA additionally revealed EE-mediated suppression of oxidative stress (GO:0006979) and apoptosis (GO:0006915) in ischemic brains (Figure 10E,F), with marked improvements in oxidative phosphorylation (GO:0006119) and intrinsic apoptosis pathways (Figure S5). Volcano plot analysis confirmed EE-induced upregulation of mitophagy regulators (*Pink1*, *Prkn*) and H_2_S synthases (*Cbs*, *Cth*), alongside enhanced *Drd1* and *Calm1* expression (Figure 10G), mechanistically linking dopaminergic signaling to CaM-mediated neuroprotection. This conclusion was further supported by in vitro experiments using cultured neurons. Compared to the OGD/R control group and the OGD/R group supplemented with exogenous H_2_S, treatment with a DR1 receptor agonist significantly enhanced the expression of DR1, calmodulin (CaM), and cystathionine γ-lyase (CSE) (Figure S6A–D), increased endogenous H_2_S levels (Figure S6E,F), and improved ATP production (Figure S6G). These findings indirectly indicate that dopamine signaling plays a regulatory role in intracellular H_2_S production, likely by acting on the DR1–CaM axis to upregulate H_2_S and its key biosynthetic enzyme expression, thereby contributing to the restoration of mitochondrial function.

## 4. Discussion

Building on existing theoretical foundations, this study is the first to demonstrate that EE facilitates endogenous H_2_S synthesis through dopaminergic signaling. This upregulation of H_2_S production synergistically activates both classical (PINK1/parkin) and non-classical (HIF-1α/BNIP3L) mitophagy pathways, thereby improving mitochondrial function and oxidative stress status in neurons following cerebral ischemia–reperfusion injury (CIRI), ultimately leading to enhanced neurological recovery. These findings integrate behavioral intervention with gasotransmitter regulation, and establish a novel neuroprotective axis—namely, the “EE–dopamine–H_2_S–mitophagy” pathway—which may offer new therapeutic strategies for post-stroke neural repair in ischemic stroke.

Current therapeutic approaches for CIRI, including antioxidant administration and exogenous H_2_S donors, are limited by their poor blood–brain barrier permeability, narrow therapeutic windows, and potential dose-dependent toxicity. In contrast, EE provides a non-invasive, systemic, and long-term safe strategy that can autonomously stimulate the neuro–endocrine–mitochondrial axis, thus overcoming the limitations of conventional treatments. In this study, supported by previous literature and transcriptomic analyses, we identified dopaminergic signaling as a crucial molecular bridge between environmental stimulation and endogenous protective responses. Specifically, EE appears to activate dopaminergic pathways—particularly via sensory and social stimulation—which in turn target the DR1-CSE/H_2_S axis [23,29], contributing to the observed neuroprotective effects.

It is noteworthy that although both classical and non-classical mitophagy pathways have been previously reported in cerebral ischemia models—for example, the pivotal role of the PINK1/parkin pathway in the clearance of damaged mitochondria has been well established [42,44], and BNIP3L-mediated mitophagy has been shown to play a critical role under hypoxic conditions [45]—there remains limited evidence regarding the role of the HIF-1α/BNIP3L pathway in cerebral ischemia [32]. The novelty of our study lies in the discovery that EE stimulation can simultaneously activate both mitophagy pathways. More importantly, we demonstrate for the first time that EE orchestrates this dual activation via upregulation of endogenous H_2_S, thereby restoring redox balance and mitochondrial homeostasis. This finding fills a critical gap in the current understanding of mitochondrial regulation in EE-based neuroprotection. EE appears to initiate a coordinated activation of the dopamine–H_2_S axis, which subsequently triggers a cascade of mitophagy-related responses. This indirect regulatory mechanism may offer a more comprehensive and physiologically balanced approach compared to direct H_2_S supplementation or modulation of individual signaling pathways.

Previous studies have established that EE activates multiple neurotrophic signaling pathways such as BDNF and VEGF [14,46], with most investigations focusing on synaptic plasticity or neurogenesis. In contrast, the present study is the first to directly link the neuroprotective effects of EE to the gasotransmitter H_2_S. H_2_S has emerged as a versatile neuromodulator capable of regulating cellular metabolic processes and maintaining homeostasis under both physiological and pathological conditions [47]. Recently, H_2_S has garnered increasing attention as a potential therapeutic agent in stroke; however, substantial gaps remain in our understanding of its precise molecular mechanisms, particularly concerning its involvement in mitophagy regulation. Although H_2_S is increasingly recognized as an endogenous antioxidant within the nervous system, its biological actions exhibit a biphasic nature. At low concentrations, H_2_S confers neuroprotection, while at high levels, it may trigger mitochondrial-dependent apoptosis via Bax activation and opening of the mitochondrial permeability transition pore (mPTP) [48]. Therefore, achieving precise spatial and temporal control of H_2_S production remains a major challenge in the field, with significant implications for balancing its therapeutic efficacy against potential cytotoxicity.

In cerebral ischemia research, novel H_2_S donors (e.g., HSDF-NH_2_) have been developed and shown to effectively reduce oxidative damage and apoptosis in ischemic models [49]. Additionally, recent investigations have utilized extracellular vesicles (EVs) derived from mesenchymal stromal cells as carriers for NaHS delivery [50], though mechanistic insights into mitochondrial actions of H_2_S remain limited. Notably, mitochondria-targeted H_2_S donors are increasingly investigated for peripheral diseases, including acute oxidative gastric mucosal injury [51], obesity-related hepatic steatosis [52], and atherosclerosis [53]. However, challenges in targeting precision, blood–brain barrier penetration, and H_2_S concentration modulation have confined cerebral ischemia–reperfusion injury studies to preliminary exploratory phases, necessitating further validation. Concurrently, redox signaling involving H_2_S and nitric oxide, particularly selective targeting of ROS-mediated pathways, has gained attention [54,55]. This study provides foundational insights for advancing H_2_S-targeted donor development and mitophagy-related mechanistic research.

At the mechanistic level, this study demonstrates, through both in vivo and in vitro models, that EE robustly activates the PINK1/parkin and HIF-1α/BNIP3L mitophagy pathways. Notably, the role of hypoxia-inducible factor 1-alpha (HIF-1α) in cerebral ischemia remains controversial. HIF-1α, a hypoxia-inducible transcription factor, is intricately associated with ROS generation [56]. Protective roles include promoting cerebral angiogenesis [57], ameliorating lipid metabolism-associated ferroptosis [58], and enhancing synaptic plasticity [59]. Contrasting evidence implicates HIF-1α in detrimental outcomes: ischemia–reperfusion injury may induce HIF-1α-mediated promoter activation of transcript release factor (PTRF), exacerbating neurological deficits [60]. Pharmacological inhibition of HIF-1α/AMP-activated protein kinase (AMPK)/Forkhead boxO 3a (FoxO3a) signaling alleviates post-ischemic ferroptosis through metabolic reprogramming [61]. Bellot et al. propose that HIF-1α enhances autophagic flux to promote cellular survival under hypoxia rather than triggering apoptosis [62]. Notably, the neurogenesis-promoting and neuroprotective capacities of HIF-1αhave been documented in prior EE studies [15]. As demonstrated by the results of this study, HIF-1α may contribute to neurological recovery after cerebral ischemia by facilitating mitophagy. This apparent contradiction in the literature suggests that the functional role of HIF-1α may be context-dependent, influenced by factors such as the timing of activation, signal intensity, and the specific cellular microenvironment. The current debate may also stem from inconsistencies across studies, including variations in experimental models, timing and mode of intervention, sample sizes, and laboratory conditions. Therefore, the precise role and underlying mechanisms of HIF-1α in CIRI remain to be fully elucidated.

A moderate level of mitophagy is essential for the timely clearance and recycling of damaged mitochondria, which in turn facilitates the biogenesis of healthy mitochondria. Conversely, disruption of this process can result in numerous detrimental cellular effects [63]. In the context of cerebral ischemia, neuronal apoptosis induced by ischemic injury has been shown to be regulated by mitophagy [64]. In this study, EE significantly enhanced mitophagy via the classical PINK1/parkin pathway. Notably, the HIF-1α/BNIP3L pathway was also upregulated under EE conditions. Recent studies have demonstrated that autophagy activation can be mediated through the HIF-1α/BNIP3 signaling axis [32], and that moderate activation of mitophagy through this pathway enables the prompt removal of damaged mitochondria, prevents the release of cytochrome c (Cyt c), and suppresses the activation of mitochondria-dependent apoptotic pathways [65], thereby preserving cellular function. Both in vivo and in vitro findings in our study support the notion that EE-induced endogenous H_2_S contributes to improved mitophagic activity.

Although NaHS appeared to further increase several mitophagy-related markers under hypoxic conditions, our data indicate that this reflects restoration of mitophagic flux rather than exacerbation of injury. The consistent reduction in p62 and the parallel improvements in cell viability, apoptotic injury, and mitochondrial integrity support the interpretation that NaHS enhances effective mitochondrial turnover, which is consistent with a compensatory normalization of mitochondrial quality control rather than an aggravation of hypoxia-induced pathology.

Several limitations of this study should be acknowledged. First, only male animals were used in the experimental models. Given the potential interaction between estrogen and H_2_S signaling, female stroke patients may exhibit different responses. The use of male mice was primarily intended to avoid confounding effects associated with hormonal fluctuations across the estrous cycle—a widely accepted strategy in preclinical ischemic stroke models [66,67]. Second, although transcriptomic profiling revealed that EE significantly modulates genes and biological pathways associated with the dopaminergic system, and our Co-IP assays and in vitro DR1 agonist experiments preliminarily suggested a mechanistic link between dopamine signaling and the H_2_S-synthesizing enzyme CSE, direct pharmacological or genetic manipulation of dopamine or its receptors was not performed in vivo. Therefore, the proposed DR1–CSE/H_2_S axis remains partially speculative and lacks definitive causal evidence to exclude the possibility of indirect regulatory mechanisms. Third, despite the integrated validation in both in vivo and in vitro models, key aspects remain unresolved, including the spatial-temporal dynamics of H_2_S within distinct neuronal subpopulations, regional differences across brain areas, and dose- or time-dependent effects. Future studies may benefit from the development of novel fluorescent probes capable of real-time, subcellular-level imaging of endogenous H_2_S. The interpretation of our bulk RNA-seq data is inherently limited by potential shifts in cellular composition after ischemic injury, such as neuronal loss or reactive gliosis. Although we minimized this variability by using uniform sampling criteria and verified comparable histological changes within groups, bulk sequencing cannot fully separate cell-type–specific contributions. These factors should be taken into account when interpreting the transcriptional differences, and future studies using single-cell or cell-type–resolved approaches will be needed to refine these observations. Moreover, the application of single-cell omics could elucidate the cell-type-specific regulatory effects of EE on neuronal subpopulations and provide deeper insight into underlying molecular pathways. It will also be essential to define the optimal therapeutic window and concentration range of H_2_S to maximize its neuroprotective effects while minimizing potential cytotoxicity. Furthermore, evaluating the generalizability of EE across sexes, age groups, and populations will enhance the translational relevance of these findings.

In summary, this study builds upon existing literature and is the first to propose that EE facilitates neuroprotection via a dopamine–H_2_S axis, which in turn activates both classical (PINK1/parkin) and non-classical (HIF-1α/BNIP3L) mitophagy pathways. This dual activation disrupts the vicious cycle between ROS accumulation and impaired mitophagy observed during cerebral ischemia–reperfusion injury. Our findings provide theoretical and experimental support for EE as a promising non-pharmacological intervention in stroke rehabilitation and suggest new molecular targets for drug development. Nevertheless, further mechanistic investigations and clinical studies are warranted to validate and expand the universality and translational potential of this neuroprotective paradigm.

## 5. Conclusions

EE exerts neuroprotective effects by activating the dopaminergic signaling pathway to enhance endogenous H_2_S biosynthesis, which ameliorates mitophagy and mitochondrial function in ischemic neurons. This cascade alleviates oxidative stress, inhibits apoptosis, and promotes functional neurological recovery.

## Data Availability

The original contributions presented in this study are included in the article/Supplementary Materials. Further inquiries can be directed to the corresponding authors.

## Supplementary Materials

The following supporting information can be downloaded at: https://www.mdpi.com/article/10.3390/antiox15010052/s1, Figure S1: Cell Viability of SH-SY5Y after treated with different concentration of NaHS; Figure S2: H_2_S-mediated mitophagy rescues mitochondrial function and oxidative stress in hypoxic-ischemic neurons. Figure S3: NAMPT and MnSOD expression profiles in peri-infarct cortical tissues of cerebral ischemic mice. Figure S4: Longitudinal body weight monitoring and Rotarod behavioral (4–40 rpm over 300 s) performance across experimental cohorts. Figure S5: GSEA plots of pathways in model versus treatment groups post RNA sequencing. Figure S6: EE may promote endogenous H_2_S production by modulating the dopamine-mediated DR1-CaM-CSE signaling pathway. Figure S7: Western blot analysis of SH-SY5Y treated with conditioned serum. Table S1: Primary antibodies used in the research.

## Abbreviations

AOAA: Aminooxyacetic acid; BNIP3L: BCL2 Interacting Protein 3-Like; CBS: cystathionine β-synthase; CSE: cystathionine γ-lyase; Drp1: Dynamin-related protein 1; DR1: Dopamine Receptor 1; DCFH-DA: 2′,7′-Dichlorodihydrofluorescein diacetate; EE: Enriched Environment; HIF-1α: Hypoxia-Inducible Factor 1α; H_2_S: Hydrogen sulfide; LC3B: Light Chain 3B of Microtubule-Associated Protein 1A/1B; MCAO: Middle cerebral artery occlusion; MDA: Malondialdehyde; MnSOD: Manganese-Superoxide Dismutase; Mfn1: Mitofusin 1; Mfn2: Mitofusin 2; mNSS: Modified Neurological Severity Score; OPA1: Optic Atrophy 1; OGD/R: Oxygen–glucose deprivation/reoxygenation; PINK1: PTEN-Induced Putative Kinase 1; parkin: Parkinson Associated E3 Ubiquitin Ligase; p-Drp1: Phosphorylated Dynamin-related Protein 1; ROS: Reactive oxygen species; TEM: Transmission electron microscope; TTC: 2,3,5-triphenyltetrazolium chloride; T-AOC: Total antioxidant capacity; YC-1: 3-(5′-Hydroxymethyl-2′-furyl)-1-benzylindazole.
