# Supplementary material for: Enriched Environment Ameliorates Cerebral Ischemia–Reperfusion Injury via Dopamine–H2S Axis-Mediated Dual Mitophagy Activation
Source: Antioxidants (Basel). 2025 Dec 30;15(1):52. doi: 10.3390/antiox15010052 (PMC12838293; doi:10.3390/antiox15010052)
Supplement: Supplementary file 1 [file antioxidants-15-00052-s001.zip › antioxidants-4005481-supplementary.pdf]

Supplementary materials

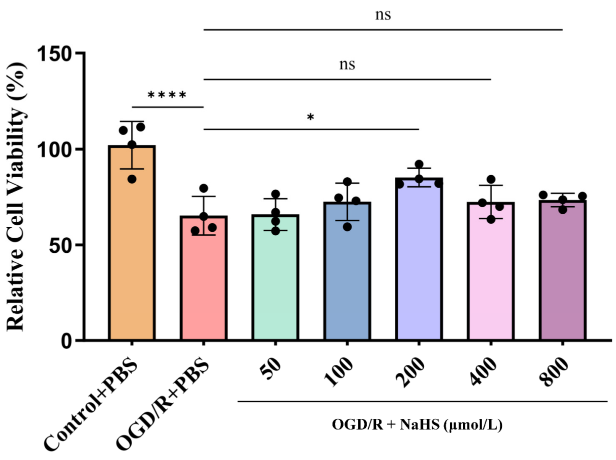

Figure S1. Cell Viability of SH-SY5Y after treated with different concentration of NaHS. Statistical significance: ns (not significant), *p < 0.05, ****p < 0.0001 (one-way ANOVA with Dunnett's multiple comparison test), n = 4.

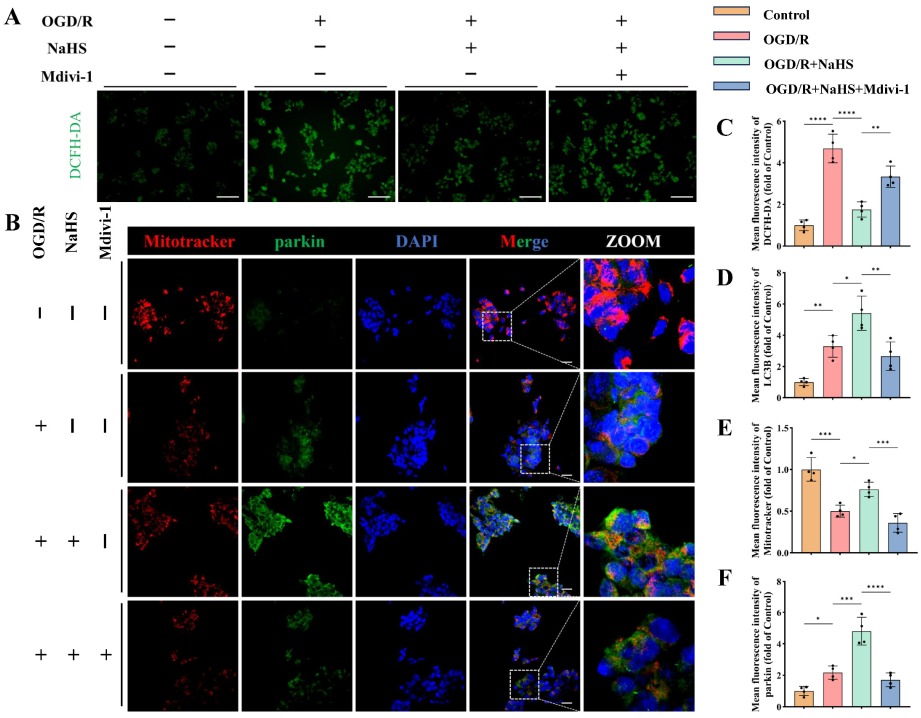

Figure S2. H_2S-mediated mitophagy rescues mitochondrial function and oxidative stress in hypoxic-ischemic neurons. (**A** and **C**) Immunofluorescence imaging of intracellular oxidative stress levels using DCFH-DA probes with corresponding

normalized quantitative analyses. Scale bar: 100 μm; $n = 4$. (**B** and **F**) Co-staining of mitochondrial-specific Mito-tracker and autophagic marker parkin, accompanied by mean fluorescence intensity quantitation. Scale bar: 20 μm; $n = 4$.（**D-E**）Quantitative analyses of Mito-tracker and LC3B co-localization corresponding to Figure 5. Statistical significance: *$p < 0.05$, **$p < 0.01$, ***$p < 0.001$, ****$p < 0.0001$, determined by one-way ANOVA (Tukey's method for multiple comparisons).

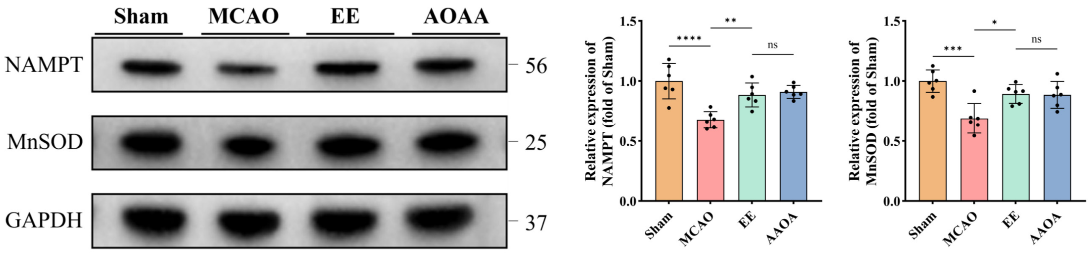

Figure S3. NAMPT and MnSOD expression profiles in peri-infarct cortical tissues of cerebral ischemic mice. Representative Western blot bands and normalized densitometric analyses of nicotinamide phosphoribosyltransferase (NAMPT) and manganese superoxide dismutase (MnSOD) expression across experimental groups. Statistical significance: ns (not significant), *$p < 0.05$, **$p < 0.01$, ***$p < 0.001$, ****$p < 0.0001$, determined by one-way ANOVA (Tukey's method for multiple comparisons), n=6.

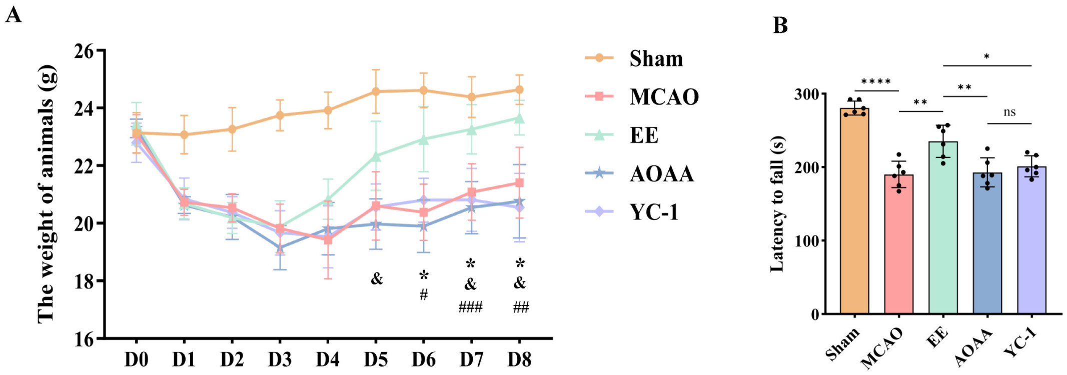

Figure S4. Longitudinal body weight monitoring and Rotarod behavioral (4-40 rpm over 300 sec) performance across experimental cohorts. Statistical significance:

ns (not significant), *$p < 0.05$, **$p < 0.01$, ****$p < 0.0001$, $n = 6$. In Picture A, * indicates the comparison between MCAO and EE groups; & indicates the comparison between EE and AOAA groups; # indicates the comparison between EE and YC-1 groups. The number of corresponding symbols represents the significance of the p-value and the difference. The differences in body weight at different time points and among different groups, as well as Rotarod behavioral performance, were analyzed using two-way ANOVA and one-way ANOVA, respectively. Tukey's test was employed for multiple comparisons.

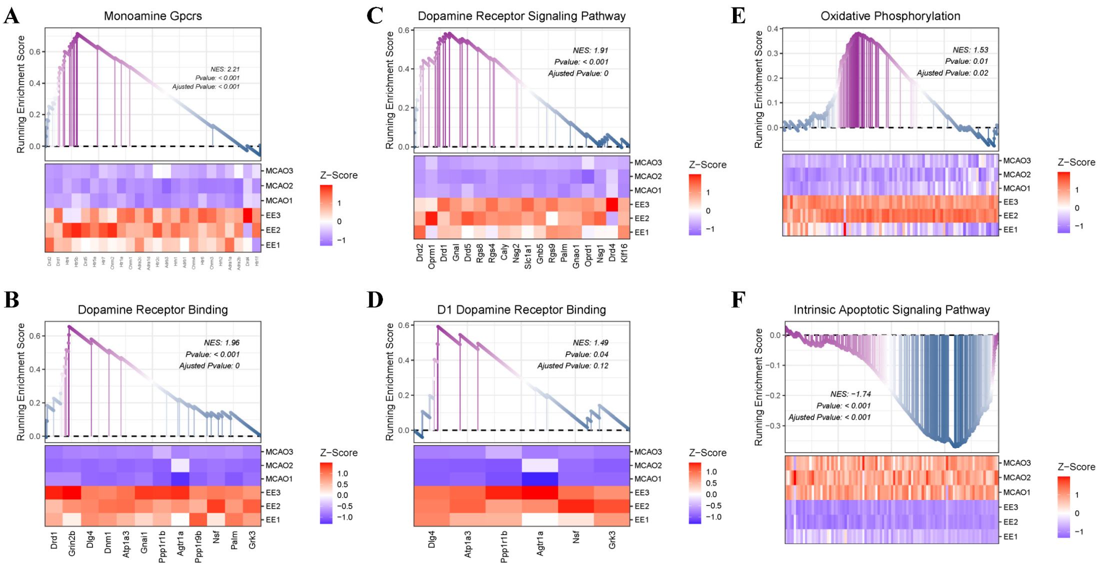

Figure S5. GSEA plots of pathways in model versus treatment groups post RNA sequencing. (**A-F**) Gene Set Enrichment Analysis (GSEA) plots illustrating transcriptomic alterations in murine cerebral tissues: Monoamine GPCR signaling (**A**), Dopamine Receptor Binding (**B**), Dopamine Receptor Signaling Pathway (**C**), D1 Dopamine Receptor Binding (**D**), Oxidative Phosphorylation (**E**), and Intrinsic Apoptotic Signaling (**F**). Data derived from high-throughput RNA-seq; $n = 3$.

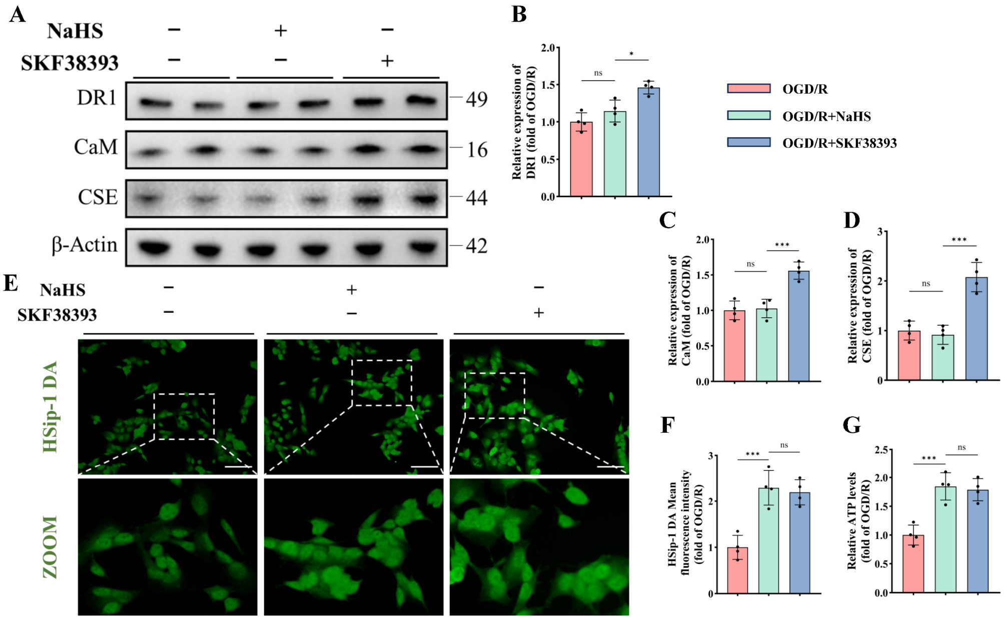

Figure S6. EE may promote endogenous H_2S production by modulating the dopamine-mediated DR1-CaM-CSE signaling pathway. (**A-D**) Representative Western blot images and quantification of DR1, calmodulin (CaM), and cystathionine γ-lyase (CSE) expression in OGD/R neuronal cultures from each group (n = 4). (**E-F**) Representative fluorescence images and quantitative analysis of H_2S levels in OGD/R neurons from each group (n = 4; scale bar: 50 μm). (**G**) ATP content in OGD/R neurons from each group (n = 4). All data are presented as mean ± standard deviation (SD). Statistical analysis was performed using one-way ANOVA followed by Tukey's post hoc test. ns: not significant; *$p < 0.05$, ***$p < 0.001$.

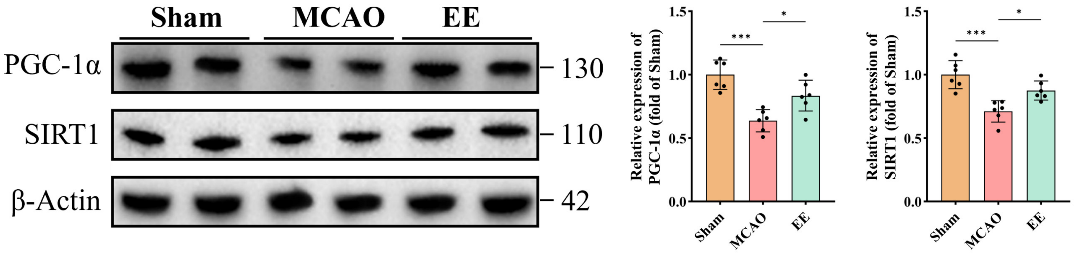

Figure S7. Western blot analysis of SH-SY5Y treated with conditioned serum. Statistical significance: *$p < 0.05$, ***$p < 0.001$, determined by one-way ANOVA (Tukey's method for multiple comparisons), n = 6.

Table S1. Primary antibodies used in the research

Antibody	Source	Catalog No.	Type	Dilution
Bcl-2	Abcam	ab182858	Rabbit mAb	1:1000(W.B.)
Bax	Abcam	ab182734	Rabbit mAb	1:1000(W.B.)
Caspase-3	Abcam	ab32351	Rabbit mAb	1:1000(W.B.)
Cleaved Caspase-3	Abcam	ab32042	Rabbit mAb	1:1000(W.B.)
DRP1	Abcam	ab184247	Rabbit mAb	1:1000(W.B.)<br>1:200(IF)
DRP1 (phospho S616)	Abcam	ab314755	Rabbit mAb	1:1000(W.B.)
OPA1	CST	80471	Rabbit mAb	1:1000(W.B.)
Mitofusin 1	Abcam	ab221661	Rabbit mAb	1:1000(W.B.)
Mitofusin 2	Abcam	ab124773	Rabbit mAb	1:1000(W.B.)
COX IV	Abcam	ab202554	Rabbit mAb	1:2000(W.B.)
Parkin	Proteintech	14060-1-AP	Rabbit pAb	1:1000(W.B.)<br>1:200(IF)
Beclin-1	CST	3495	Rabbit mAb	1:1000(W.B.)
SQSTM1/p62	CST	39749	Rabbit mAb	1:1000(W.B.)
LC3B	Affinity	AF4650	Rabbit pAb	1:1000(W.B.)<br>1:200(IF)
NAMPT	CST	86634	Rabbit mAb	1:1000(W.B.)
SOD2/MnSOD	CST	13141	Rabbit mAb	1:1000(W.B.)
BNIP3L	Affinity	DF8163	Rabbit pAb	1:1000(W.B.)
PINK1	Affinity	DF7742	Rabbit pAb	1:1000(W.B.)
HIF-1α	ThermoFisher	700505	Rabbit mAb	1:1000(W.B.)<br>1:200(IF)
DR1	GENE TEX	GTX55597	Rabbit pAb	1:1000(W.B.)
Calmodulin/CaM	Proteintech	10541-1-AP	Rabbit pAb	1:1000(W.B.)<br>2.0μg(IP)
Gamma Cystathionase/CSE	Proteintech	12217-1-AP	Rabbit pAb	1:1000(W.B.)<br>2.0μg(IP)
CBS	Proteintech	14787-1-AP	Rabbit pAb	1:1000(W.B.)
PGC-1α	CST	2178	Rabbit mAb	1:1000(W.B.)
SIRT1	CST	9475	Rabbit mAb	1:1000(W.B.)

β-Actin	Abcam	ab8226	Mouse mAb	1:5000(W.B.)
GAPDH	Abcam	ab8245	Mouse mAb	1:5000(W.B.)
α-Tubulin	Abcam	ab7291	Mouse mAb	1:5000(W.B.)
